# Interleukin-36 is overexpressed in human sepsis and IL-36 receptor deletion aggravates lung injury and mortality through epithelial cells and fibroblasts in experimental murine sepsis

**DOI:** 10.1186/s13054-023-04777-z

**Published:** 2023-12-13

**Authors:** Huachen Wang, Meixiang Wang, Junlan Chen, Hongda Hou, Zheng Guo, Hong Yang, Hua Tang, Bing Chen

**Affiliations:** 1 Institute of Infectious Diseases, The Second Hospital of Tianjin Medical University, 23 Pingjiang Road, Tianjin, 300211 People’s Republic of China; 2 Intensive Care Unit, The Second Hospital of Tianjin Medical University, Tianjin, China; 3 The First Affiliated Hospital of Shandong First Medical University, Jinan, China; 4 State Key Laboratory of Oral and Maxillofacial Reconstruction and Regeneration, Key Laboratory of Oral Biomedicine Ministry of Education, Hubei Key Laboratory of Stomatology, School and Hospital of Stomatology, Wuhan University, Wuhan, Hubei China; 5 Division of Epidemiology, Department of Medicine, Vanderbilt University Medical Center, 2525 West End Ave, Nashville, TN USA; 6 The Province and Ministry Co-Sponsored Collaborative Innovation Center for Medical Epigenetics, Department of Pharmacology, School of Basic Medical Sciences, Tianjin Medical University, No. 22 Qixiangtai Road, Heping District, Tianjin, China; 7 Department of Rheumatology and Autoimmunology, Shandong Provincial Key Laboratory for Rheumatic Disease and Translational Medicine, The First Affiliated Hospital of Shandong First Medical University and Shandong Provincial Qianfoshan Hospital, Jinan, 250014 Shandong China; 8 Institute of Infection and Immunity, Medical Science and Technology Innovation Center, Shandong First Medical University and Shandong Academy of Medical Sciences, Jinan, 250000 Shandong China

**Keywords:** Sepsis, Inflammation, IL-36, IL-36R, Mortality

## Abstract

**Background:**

Sepsis is defined as a life-threatening syndrome caused by an unbalanced host response to infection. The role of interleukin (IL)-36 cytokines binding to the IL-36 receptor (IL-36R) in host response during sepsis remains unknown.

**Methods:**

Serum IL-36 level was measured in 47 septic patients sampled on the day of intensive care unit (ICU) and emergency department admission, 21 non-septic ICU patient controls, and 21 healthy volunteers. In addition, the effects of IL-36R deletion on host inflammatory response in cecal ligation and puncture (CLP)-induced polymicrobial sepsis was determined.

**Results:**

On the day of ICU and emergency department admission, the patients with sepsis showed a significant increase in serum IL-36 levels compared with ICU patient controls and healthy volunteers, and the serum IL-36 levels were related to the severity of sepsis. Non-survivors of septic patients displayed significantly lower serum IL-36 levels compared with survivors. A high serum IL-36 level in ICU and emergency department admission was associated with 28-day mortality, and IL-36 was found to be an independent predictor of 28-day mortality in septic patients by logistic regression analysis. Furthermore, IL-36R deletion increased lethality in CLP-induced polymicrobial sepsis. Septic mice with IL-36R deletion had higher bacterial load and demonstrated more severe multiple organ injury (including lung, liver, and kidney) as indicated by clinical chemistry and histopathology. Mechanistically, IL-36R ligands released upon lung damage activated IL-36R^+^lung fibroblasts thereby inducing expression of the antimicrobial protein lipocalin 2. Moreover, they induced the apoptosis of lung epithelial cells.

**Conclusions:**

Septic patients had elevated serum IL-36 levels, which may correlate with disease severity and mortality. In experimental sepsis, we demonstrated a previously unrecognized role of IL-36R deletion in increasing lethality.

**Supplementary Information:**

The online version contains supplementary material available at 10.1186/s13054-023-04777-z.

## Background

Sepsis, a pathological condition caused by an unregulated response to pathogen invasion [1], is a major global cause of mortality with no effective treatments [2]. In sepsis, the host’s immunity fails to eradicate invading pathogens, leading to immune suppression and abnormal inflammation [3–5]. Infection control and organ function support are the mainstays of sepsis treatment [6, 7], but no specific agents are currently approved for the treatment of sepsis [8, 9]. Therefore, we need to understand sepsis thoroughly and discover new treatment strategies.

Interleukin (IL) -36 cytokines, a member of the IL-1 cytokines family(IL-1, IL-33, IL-36 IL-37, IL-38, and IL-1Ra), includes three agonists IL-36α, IL-36β, IL-36γ, and an antagonist IL-36Ra [10]. IL-36 agonists bind to the same receptor complex, which is composed of the IL-36 receptor (IL-36R or named IL1RL2) and IL-1 receptor accessory protein (IL-1RacP) [11]. IL-36 and IL-36R are widely expressed in a variety of tissues, including the lung, skin, and gut, and IL-36 signaling regulates inflammatory and immune responses in these tissues [12–14]. Bronchial epithelial cells upregulate IL-36 expression following stimulation of pro-inflammatory cytokines, and bacterial and virus-associated molecular patterns [15]. Intratracheal injection of IL-36α or IL-36γ in mice induces pro-inflammatory cytokines, chemokines, and neutrophils recruitment [16]. However, the role mechanism of IL-36 signaling in the pathogenesis of lung inflammation induced by sepsis is unknown.

In this study, we aimed to determine the extent of IL-36 expression in septic patients and investigate the relationship between IL-36R and disease severity and survival in sepsis. Levels of IL-36 isoforms are elevated in septic patients and are inversely correlated with related inflammatory markers such as procalcitonin (PCT). As compared with wild-type (WT) controls, IL-36R^−/−^ mice display increased inflammatory cells, bacterial load, and decreased antimicrobial protein, which is associated with increased mortality in response to sepsis challenge. Taken together, these data demonstrate for the first time the important role of IL-36R ligands in the pathogenesis of sepsis-induced lung injury.

## Materials and methods

### Patients and controls

From October 2021 to October 2022, every consecutive patient admitted to the emergency Intensive Care Unit and the emergency department of The Second Hospital of Tianjin Medical University, Tianjin, China, was prospectively enrolled. Patients who met the clinical criteria for The Third International Consensus Definitions for Sepsis and Septic Shock (Sepsis-3) were screened for eligibility within the first day [17]. The exclusion criteria were as follows: (1) patients diagnosed with a malignant tumor, organ transplantation, chronic viral infection (hepatitis, AIDS), chronic renal insufficiency, autoimmune diseases, and immunosuppressive drugs in the past 8 weeks; (2) age < 18 years or pregnancy; (3) transfer from another ICU were excluded from the study.

Forty-seven patients with sepsis and twenty-one non-septic patients were enrolled in this study. Non-septic patients in critical condition who were admitted to the ICU were recruited as ICU controls; the diagnoses of the ICU control patients included acute heart failure (n = 9), poly-trauma (n = 4), burns (n = 5) and hypertensive emergencies (n = 3). The clinical data, including Sequential Organ Failure Assessment (SOFA) score, the levels of procalcitonin (PCT) and C-reaction protein(CRP), or mortality during the 28-day, were recorded.

In addition, 21 healthy donors who had no medical problems in the medical examination center of The Second Hospital of Tianjin Medical University served as healthy controls. This protocol was approved by the Clinical Research Ethics Committee of the Second Hospital of Tianjin Medical University (KY2022K234), and informed consent was conformed to the Declaration of Helsinki [18]. The consent of each participant was obtained at admission. If the patient has lost consciousness or capacity, written informed consent was then obtained from each enrolled patient’s nearest relative or designated person.

### Sepsis model

IL36R^−/−^ mice were C57BL/6 background and purchased from Cyagen Biosciences (Suzhou, China). In all experiments, age and sex-matched littermate controls were used. Specific pathogen-free 6–8-week-old female mice were obtained from and raised at Shandong First Medical University and Shandong Academy of Medical Sciences. Polymicrobial sepsis was caused by cecal ligation and puncture (CLP) as described in previous studies [19, 20]. All operations were carried out under pentobarbital sodium anesthesia, and every effort was made to minimize the pain of mice. The cecum is ligated at half of the distance between the distal end and the base of the cecum. And then, the cecum was then punctured once with a 22-gauge needle. The cecum was then returned to the abdominal cavity and the incision was sutured. Sham-operated (control) animals underwent identical laparotomy; the cecum was returned to the abdominal cavity without ligation or puncture. Mice were resuscitated by subcutaneous injection of saline (5 ml per 100 g body weight). Survival was monitored twice daily for 7 days. All studies were approved by the local Animal Care and Use Committee.

### Cytokine measurement

For all participator, the WBC (white blood cell), PCT (procalcitonin), CRP (C-reaction protein), and other indicators were detected on the day of enrollment. At the same time, venous blood samples were collected from the patients, and then, the serum were isolated and frozen at − 80 °C until IL-36 subtypes were assayed. After 3 or 7 days of CLP in mice, we collected and stored serum using the methods described above. For measurement of cytokines in mouse lung, we triturated and centrifuged lung tissue [100 mg tissue/1 ml phosphate buffer saline (PBS)] to obtain the supernatant.The concentration of human (mouse) IL-36 was determined by a human (mouse) IL-36 ELISA kit (Jiangsu Meimian Industrial Co., Ltd., MM-50540H1 MM-50542H1 MM-50544H1 MM-50540H2 MM-50542H2 MM-50544H2). Human (mouse) IL-1Ra ELISA kit from the same manufacturer as the above kits (Jiangsu Meimian Industrial Co., Ltd., MM-2793H1 MM-1003M1).

### Serum biochemistry

Alanine aminotransferase (ALT), aspartate transaminase (AST), urea, and creatinine were measured with commercially available kits using a Hitachi analyzer (Boehringer Mannheim, Mannheim, Germany) according to the manufacturers’ instructions.

### Determination of bacterial colony-forming unit (CFU)

Mice were sacrificed at specific time points following CLP. The lungs, livers, kidneys, hearts, and spleens were isolated, and 10 mg of each tissue was homogenized in 700 μl PBS. A total of 10 μl of each dilution was separately plated on blood-agar plates. Colony-forming unit (CFU) numbers were counted after 24 h of incubation at 37 °C.

### Histopathology and immunofluorescence

Lungs were removed from euthanized mice and fixed in 4% formalin, stained with hematoxylin–eosin, and examined with microscope. To score lung inflammation and damage, the entire lung surface was measured using the lung injury scoring system as described [21]. Lung injury scoring system parameters include neutrophils in the alveolar space (A), neutrophils in the interstitial space (B), hyaline membranes (C), proteinaceous debris filling the airspaces (D), and alveolar septal thickening (E). At least 20 random areas were scored blindly and independently as 0–2 at × 400 magnification. The sum of scores for all parameters represents the total pathology score for the lung.

Primary antibodies for stainings of mouse tissue were: mouse IL-36R antibody (DF8987, Affinity), mouse antihuman epithelial cell adhesion molecule (EPCAM) (GB11274, Servicebio), anti -PDGFR alpha Rabbit (GB111342, Servicebio), anti -Lipocalin-2/NGAL Rabbit (GB111134, Servicebio), and anti-S100A9 Rabbit (GB111149, Servicebio).

### RNA isolation and real-time PCR

Total RNA extracted from lung tissue was reverse transcribed. Gene expression was measured in the LightCycler/LightCycler 480 System (Roche Diagnostics, USA) using the SYBR Realtime PCR Kit (Takara, Japan). GAPDH served as the internal reference. Mean Ct values for target gene analyses were normalized to the mean Ct values of GAPDH. Primer sequences were synthesized by General Biotech Co., Ltd (Shanghai, China) (Additional file 11: Table S2).

### RNA sequencing and data analysis

Total RNA of epithelial cell (1 × 10^6^ cells/sample) isolated from lung tissue was extracted using RNeasy mini kit (Qiagen, Hilden, Germany). RNA quality was determined and quantified by a 2100 Bioanalyzer (Agilent). RNA sequences were executed on the Illumina HiSeq 2500 platform at Berry Genomics Company (Beijing, China).

### BM chimeric mice

Approximately 6- to 8-week-old WT or IL-36R^−/−^ recipient mice were lethally irradiated with two doses of 5.5 Gy, each separated by approximately 4 h. WT or IL-36R^−/−^ mice as donors were sacrificed and bone marrow (BM) cells from femurs and tibias were harvested under sterile conditions. Following red blood cell depletion with Red Blood Cell Lysis Buffer, approximately 2 × 10^6^ unfractionated BM cells in 200 µl PBS were injected into the tail vein of each recipient mouse. Four groups of IL-36R^−/−^ chimera mice were generated: WT → WT, IL-36R^−/−^  → WT, WT → IL-36R^−/−^, and IL-36R^−/−^  → IL-36R^−/−^. BM chimeric mice were subjected to CLP at 8 weeks post BM cell transfer.

### Lung cell isolation and flow cytometry

For isolating fibroblasts and epithelial cells, mouse lung tissues were digested with Collagenase IV (1 mg/mL, Sigma) and DNase I (0.05 mg/mL, Roche) in HBSS for 60 min at 37 °C with several interval vortexes. Lung cell suspensions were incubated with antibodies including CD45, CD31, IA/IE, LYVE1, CD326, PDGFRα, and Ter119. Cell sorting was performed with a FACS Aria III cell sorter (Becton Dickinson) for subsequent experiments.

The cells were sequentially gated using the following makers: macrophages(CD45^+^CD64^+^MerTK^+^),neutrophils(CD45^+^Ly6G^+^), natural killer cells(CD64^−^MerTK^−^CD11b^−^Ly6G^−^ NK1.1^+^), dendritic cells (CD64^−^MerTK^−^CD11b^−^Ly6G^−^ NK1.1^−^IA/IE^+^ CD11c^+^), T cells (CD64^−^MerTK^−^CD11b^−^Ly6G^−^NK1.1^−^IA/IE^−^CD11c^−^CD3^+^), B cells (CD64^−^MerTK^−^CD11b^−^Ly6G^−^NK1.1^−^IA/IE^−^CD11c^−^CD19^+^), epithelial cells (CD45^−^CD31^−^LYVE1^−^IA/IE^−^Ter119^−^CD326^+^), endothelial cells (CD45^−^LYVE1^−^IA/IE^−^Ter119^−^CD326^−^CD31^+^), fibroblasts (CD45^−^CD31^−^LYVE1^−^IA/IE^−^Ter119^−^CD326^−^PDGFRα^+^). Cell late apoptosis was defined as Annexin V and 7-AAD double-positive cells. Cell early apoptosis was defined as Annexin V positive and 7-AAD negative cells. Cell death was defined as 7-AAD positive and Annexin V negative cells. Flow cytometry analysis was performed using a FACS ARIA II (BD Biosciences) equipped with a 405 nm, 488 nm, and 633 nm laser, and the results were analyzed by FlowJo 7.6 software (Tree Star, OR, USA, RRID: SCR_008520). Antibodies are listed in the Additional file 12: Table S3.

### Public data acquisition

The GEO database (https://www.ncbi.nlm.nih.gov/geo/) was searched using the search formula “((Expression profiling by array[Filter]) AND Homo sapiens [Organism]) AND whole blood samples AND sepsis”. One suitable dataset, GSE69063, was obtained. The GEO database was searched using the search formula “((Expression profiling by high throughput sequencing [Filter]) AND Mus musculus [Organism]) AND sepsis”. One suitable dataset, GSE179554, was obtained. The GEO database was searched using the search formula “(((Expression profiling by high throughput sequencing [Filter]) AND Mus musculus [Organism]) AND sepsis) AND lung”. One scRNAseq dataset, GSE207651, was obtained. The GSE69063, obtained using the GPL19983 platform, including healthy samples and sepsis samples from the emergency department (ED). Samples were collected at ED arrival (T0), 1 h later (T1), and 3 h post arrival (T2). The GSE179554 dataset, obtained using the GPL24247 platform, contains gene expression information of four important organs (kidney, lung, liver, and heart) under sepsis. The GSE207651, obtained using the GPL19087 platform, contains gene expression information of CD45^−^ and CD45^+^ cell suspensions in lung.

### Differentially expressed gene screening and correlation analysis in RNA-seq data

The limma package in R language was used for differential expression analysis of genes. Gene expression values of all samples were normalized using the limma package 45. The differentially expressed genes in above datasets and our own dataset were analysed between the control group and sepsis group. The screening criteria of differentially expressed genes were | log2FC |> 1 and adjusted *P* value < 0.05. The pheatmap R package (1.0.12) was used to produce heatmaps of differentially expressed genes (DEGs) between groups. The correlation function of the R language can be used to calculate the correlation coefficient between differentially expressed genes.

Enrichment analysis of differentially expressed genes in RNA-seq data Gene Ontology (GO) analysis and Genomes (KEGG) analysis of differentially expressed genes in our RNA-seq dataset. GO enrichment analysis of differentially expressed genes were implemented by the topG (http://www.bioconductor.org/packages/release/bioc/html/topGO.html), in which gene length bias was corrected. GO terms with corrected p-values less than 0.05 were considered significantly enriched by differential expressed genes. The most enriched 20 GO terms were presented. ClusterProfiler R package (3.14.3) was used to perform the KEGG pathway enrichment analysis. KEGG pathways with adjusted *p* values < 0.05 were considered statistical significance in the enrichment analysis. The most enriched 20 KEGG pathways were presented.

### General analyses of single-cell sequencing data

The GSE207651 datasets were analysed using ‘Seurat’ v4.1.1 in R [22]. Genes expressed in less than 10 cells were excluded from subsequent analysis. The following cells were excluded from subsequent analysis: (1) percent of mitochondrial genes was more than 5%; (2) number of read counts was less than 2000; and (3) number of feature genes was less than 200. The raw count of each cell is normalized by multiplying the total number in that cell by one million, and then by adding a pseudo-count of 1 for a natural logarithmic transformation. The top 2000 high-variant genes (HVG) were selected based on average expression and variance. Seurat objects are scaled by percentage of mitochondrial genes and sample identity. The first 50 principal components were calculated out of 2000 HVGs, and the principal component dimensionality reduction was carried out. An algorithm based on shared nearest neighbor (SNN) was used to identify cell clusters. The main cell classes were identified by enrichment of EPCAM (epithelial), PECAM1 (endothelial), PTPRC (immune), PDGFRA/PDGFRB/ACTA2 (fibroblast) genes.The Wilcox test implemented in Seurat was used to detect differential gene expression between clusters. Top 5 differential genes in fibroblasts and epithelial cells from our current study were demonstrated.

### Western blot

The epithelial cell (1 × 10^6^ cells/sample) isolated from lung cell suspensions with or without 3 days' CLP. The cells were lysed with RIPA buffer (Thermo Fisher Scientific, Waltham, MA, USA) supplemented with Halt protease and phosphatase inhibitor cocktail (Thermo Fisher Scientific, Waltham, MA, USA), and the total protein concentration was quantified by the Bradford assay kit (Coomassie Plus, Thermo Fisher Scientific, Waltham, MA, USA) and adjusted accordingly. The proteins were separated by SDS-PAGE and transferred to a PVDF membrane (Immobilon FL, Millipore, Billerica, MA, USA). The membranes were blocked and blotted with primary antibodies against β-actin (Beyotime, #AF5001, 1:100 dilution), p65 (Cell Signaling Technology, #8242S,1:1000 dilution) and p-p65 (Bioss, #bs-0982,1:1000 dilution) at 4 °C overnight. HRP (Horseradish peroxidase) conjugated goat anti-mouse (Beyotime, #A026) or anti-rabbit Ig G (Beyotime, #A0208) were used as secondary antibodies. They were then blotted with HRP for 1 h at room temperature. Band densities were quantified using protein simple software and ImageJ.

### Statistical analysis

Human data were expressed as scatter dot plots with medians. Mice and cell data were expressed as mean ± standard deviation (SD). The student’s *t* test was applied for normal distribution data. The Mann–Whitney U test or Kruskal–Wallis test followed by Dunn’s multiple comparisons post-test was performed for non-normal distribution data. To test correlations between two parameters, a non-parametric Spearman’s rank correlation coefficient was used.To determine the discriminative power of IL-36 for 28-day mortality and diagnosis, we plotted multiple sets of receiver-operating characteristic (ROC) curves by selecting different data sets in column analyses, and calculated the area under the curve (AUC) with its 95% confidence interval (CI) by GraphPad Prism. For murine survival studies, Kaplan–Meier analyses followed by log-rank tests were performed. All analyses were done using GraphPad Prism version 7.0 (GraphPad Software, San Diego, CA). p values less than 0.05 were considered statistically significant.

## Results

### Sepsis resulted in elevated serum IL-36 levels

**Fig. 1:**
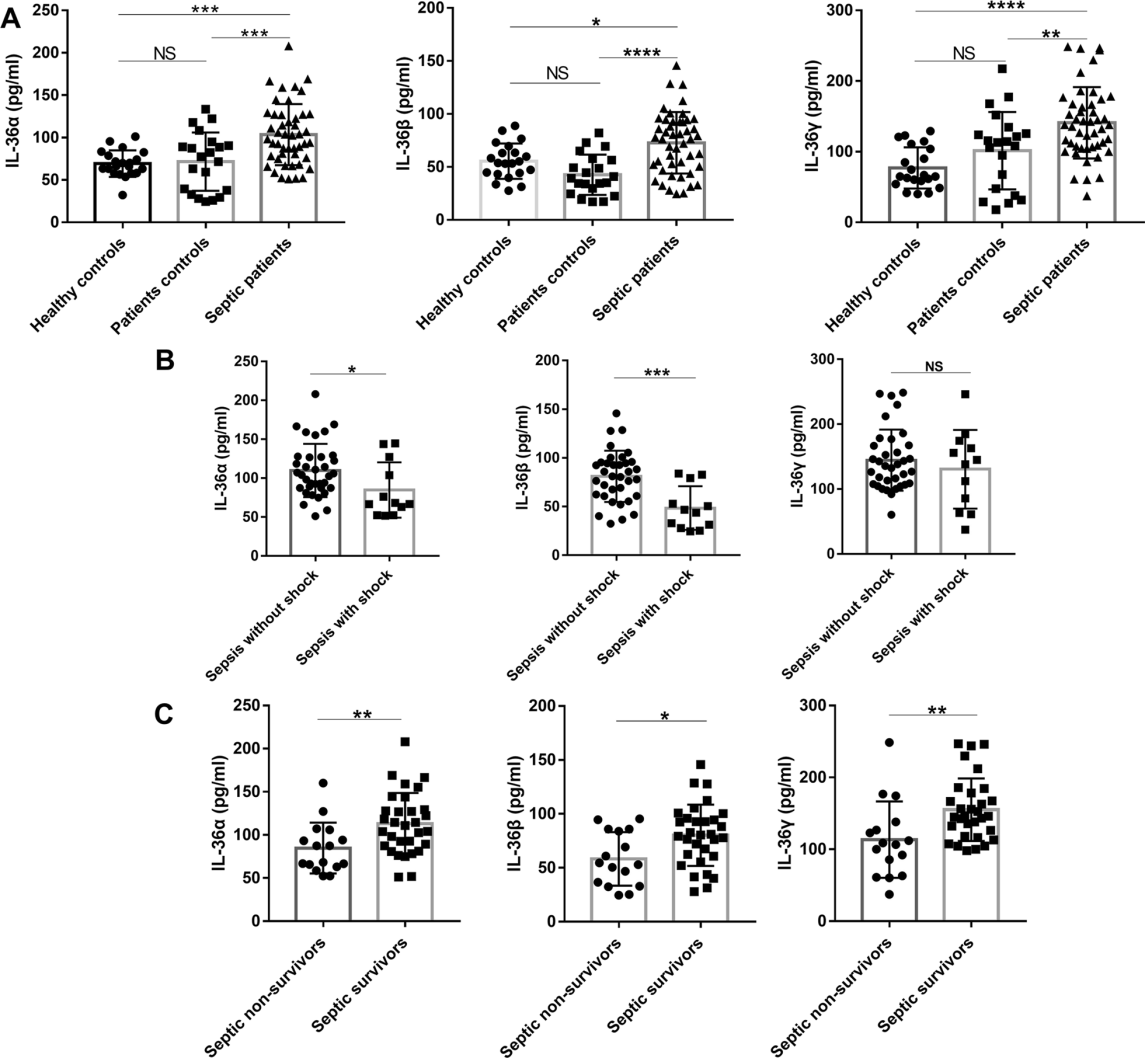
Serum interleukin (IL)-36 levels at admission were elevated in the patients with sepsis. **A** Interleukin (IL)-36 subtypes concentrations were measured by ELISA in serum samples collected from 47 patients with sepsis, 21 non-septic ICU controls, and 21 healthy control subjects. **B** IL-36 subtypes concentrations in serum samples collected from septic shock patients and patients without shock. **C** IL-36 subtypes concentrations in serum samples collected from survivors and non-survivors in the patients with sepsis. Non-parametric Mann–Whitney U test or Kruskal–Wallis test followed by Dunn’s multiple comparisons post test was used to compare results between groups. Data are means ± SD, error bars represent SD and dots represent individual participants. *NS* not significant; *P* ≤ 0.05 were considered statistically significant. *P < 0.05; **P < 0.01; ***P < 0.001; ****P < 0.0001

IL-36R exhibited markedly higher expression in human blood than healthy controls according to the published databases (Additional file 1: Fig. S1). IL-36 subtypes (IL-36α, IL-36β, and IL-36γ) in sepsis patients were significantly increased or tended to increase at different time points. To further study the role of the IL-36 signal, we collected serum from sepsis patients, ICU patient controls, and healthy controls. Clinical characteristics of sepsis patients, ICU patient controls, and healthy controls were presented in Additional file 10: Table S1. In the serum of 47 sepsis patients on the day of admission, IL-36 subtypes levels were significantly higher than patient controls and healthy individuals (Fig. 1A). Compared with sepsis patients without shock, the serum IL-36α and IL-36β concentrations of septic shock patients at admission were significantly decreased (Fig. 1B). The IL-36γ level of septic shock patients at admission was also decreased. However, it did not reach a statistical difference (Fig. 1B). The 28-day mortality was 34.0% in these septic patients. Sepsis non-survivors showed significantly lower IL-36 levels than sepsis survivors (Fig. 1C).

### The clinical role of IL-36 in diagnosing sepsis and predicting 28-day mortality

**Fig. 2:**
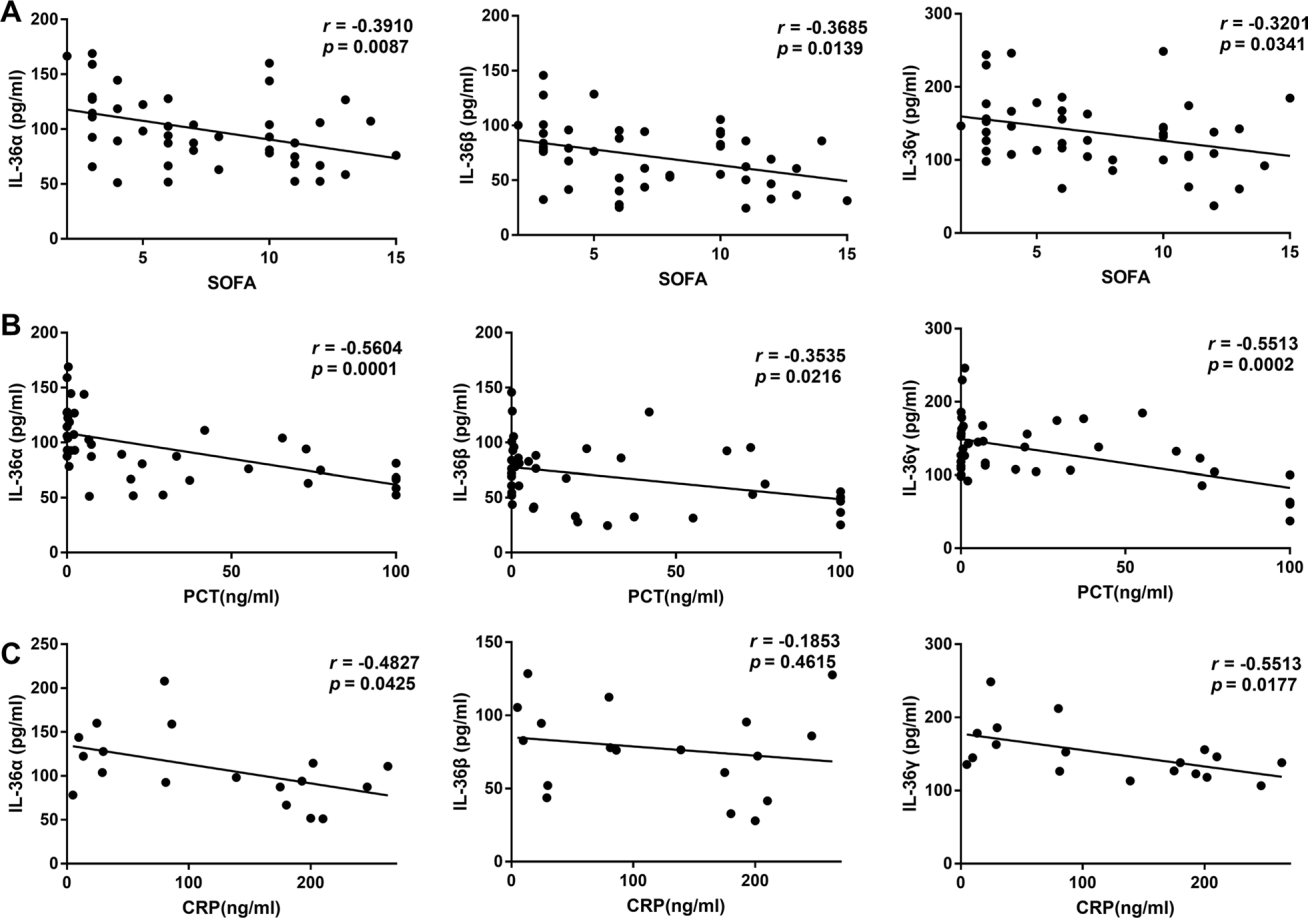
Serum interleukin (IL)-36 subtypes levels at admission correlated with disease severity in septic patients. **A** Correlation of interleukin (IL)-36 subtypes levels with Sequential Organ Failure Assessment (SOFA) scores in the patients with sepsis. **B** Correlation of IL-36 subtypes levels with procalcitonin (PCT) levels in the patients with sepsis. **C** Correlation of IL-36 subtypes levels with C-reactive protein (CRP) levels in the patients with sepsis. Spearman’s correlation coefficient was used to evaluate the correlation between concentrations of IL-36 subtypes and SOFA scores, PCT, or CRP levels. Dots represent individual participants

Higher serum IL-36 subtypes levels were related to lower SOFA scores in the patients with sepsis on the day of admission (Fig. 2A). Moreover, there was a significant correlation between IL-36 subtypes and PCT (Fig. 2B), or CRP (Fig. 2C) levels on admission.

The ROC curve of IL-36 subtypes for diagnosing sepsis is shown in Additional file 2: Fig. S2. The AUC of IL-36α on the day of admission was 0.797 (*p* < 0.001, [95% CI] 0.693–0.797). The AUC of IL-36β on the day of admission was 0.681 (*p* = 0.005, [95% CI] 0.555–0.681). The AUC of IL-36γ on the day of admission was 0.863 (*p* < 0.001, [95% CI] 0.776–0.863).

In addition, we analyzed whether each IL-36 isoform contributes to predicting 28 days in septic patients. For the prediction of 28-day mortality (Additional file 3: Fig. S3), the area under the ROC curve for IL-36α on the day of admission was 0.730 (*p* = 0.004, [95% CI] 0.572–0.730), higher than the AUC for IL-36β (AUC = 0.706; [95% CI] 0.547–0.706, *p* = 0.011) but lower than the AUC for IL-36γ (AUC = 0.756; [95% CI] 0.589–0.756,* p* = 0.003) and SOFA score (AUC = 0.738; [95% CI] 0.578–0.897,* p* = 0.011).

### IL-36 receptor deletion aggravated CLP-induced sepsis mortality

To investigate whether levels of IL-36 family members are elevated in septic mice, we generated a sepsis model and examined protein expression levels of IL-36α, IL-36β, and IL-36γ during sepsis. Both IL-36α, IL-36β, and IL-36γ levels were significantly elevated in serum and lungs at 3-day and 7-day post-CLP challenge (Additional file 4: Fig. S4). The above data suggest that IL-36 family member's production is also significantly increased during mouse sepsis, indicating that IL-36 signaling may be involved in the pathogenesis of sepsis. IL-1Ra, an anti-inflammatory cytokine in IL-1 family, serum levels were also significantly elevated in septic patients and mouse (Additional file 5: Fig. S5).

**Fig. 3:**
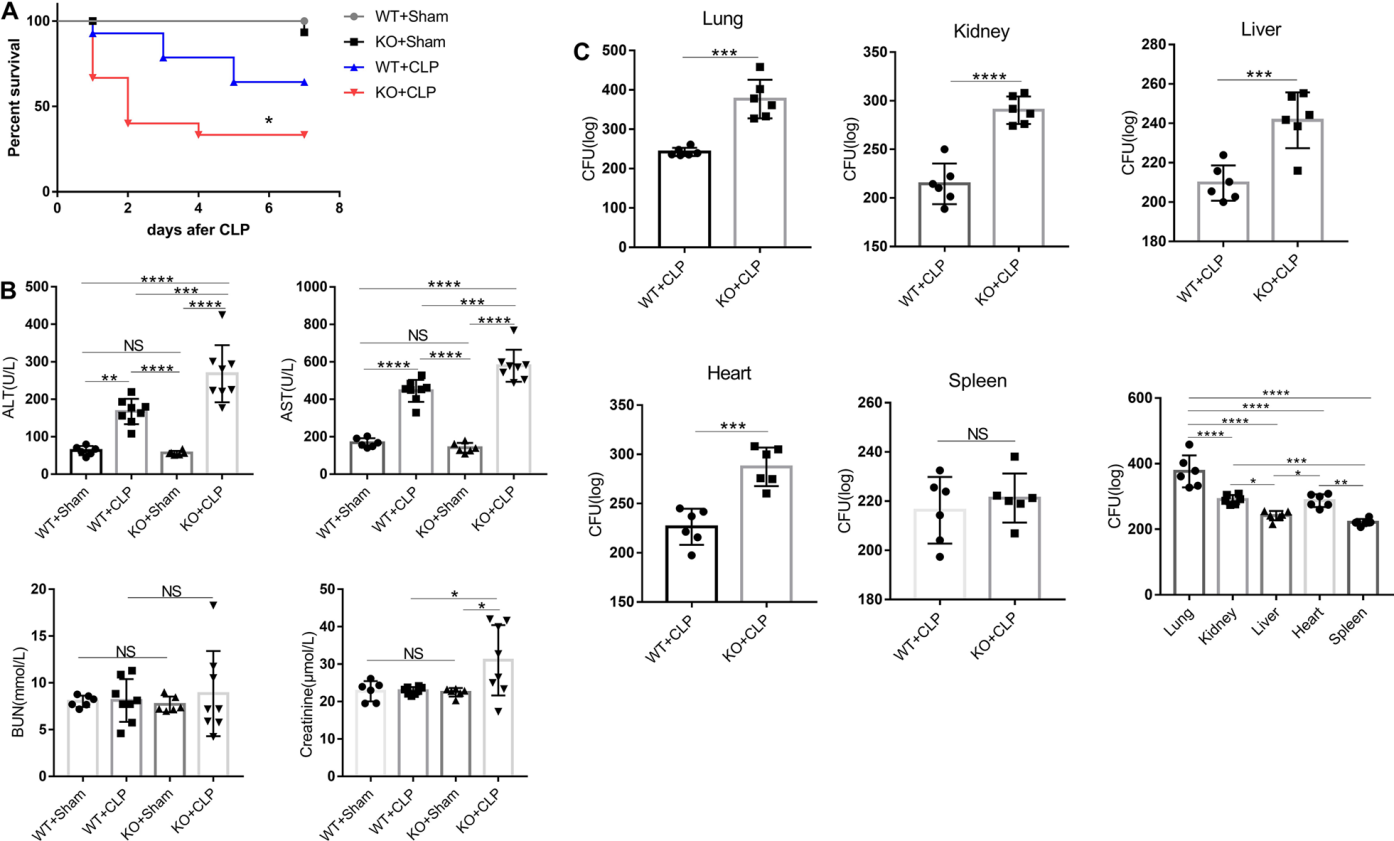
Interleukin (IL)-36R-signalling afforded protection against sepsis. **A** Survival of cecal ligation and puncture (CLP) and sham operation mice (n = 16 per group) after interleukin (IL)-36R knock out or not. Comparison between groups was done by Kaplan–Meier analysis followed by log-rank tests. **B** Serological markers of organ injury including alanine aminotransferase (ALT), aspartate aminotransferase (AST), blood urea nitrogen (BUN), and creatinine in interleukin (IL)-36R knock out mice at 3 days after CLP. Statistical difference was denoted by the horizontal bracket (Mann–Whitney U test). **C** Vital organs homogenates from CLP mice with or without interleukin (IL)-36R knock out were cultured on blood agar plates, and the number of bacterial colonies was counted as colony-forming unit (CFU). Statistical difference was denoted by the horizontal bracket (Mann–Whitney U test). Three independent experiments were performed thrice. Data are means ± SD and error bars represent SD. *P* ≤ 0.05 were considered statistically significant. *P < 0.05; **P < 0.01; ***P < 0.001; ****P < 0.0001

IL-36α, IL-36β, and IL-36γ all bind only to the IL-36 receptor (IL-36R). To examine whether IL-36R mediated signaling is involved in the host response against CLP-induced polymicrobial sepsis, WT and IL-36R^−/−^ survival were assessed out to 7 days. IL-36R deletion dramatically decreased septic mouse survival compared with the WT septic mice (Fig. 3A).

Exacerbated mortality of IL-36R^−/−^ septic mice was closely associated with increased organ injury. Serum concentrations of ALT and AST, markers for hepatocellular injury, and creatinine, a marker for renal failure, were significantly increased in IL-36R^−/−^ septic mice at 3 days after CLP (Fig. 3B).

To determine whether there is any difference in bacterial clearance, the homogenate diluents of lungs, livers, kidneys, hearts, and spleens were cultured on blood agar plates. Vital organs bacterial CFU levels were significantly increased in the IL-36R^−/−^ mice compared to WT mice (Fig. 3C). And CFU levels in the lung were higher than that in other organs (Fig. 3C).

IL-36R is predominantly expressed in the lungs of mice by analyzing a published RNA-Seq dataset (GSE179554) (Additional file 6: Fig. S6A). The expression of IL-36R ligands was also analysed by quantitative PCR in heart, kidney, liver, and lung (Additional file 6: Fig. S6B).

**Fig. 4:**
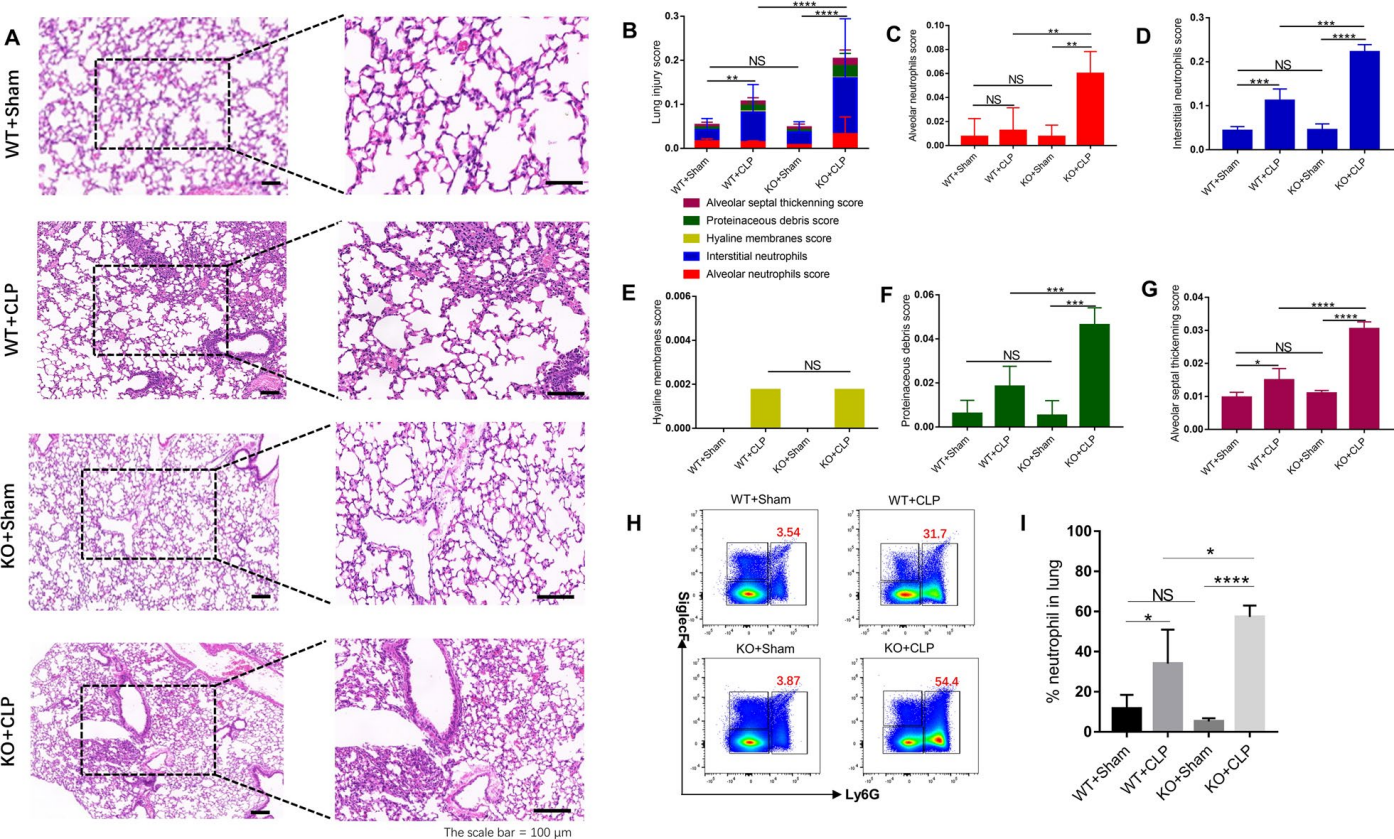
The protective effect of interleukin (IL)-36R on lung injury in CLP mice. **A** The histological images of H&E stained lung sections; the scale Bar = 100 μm. **B** Total lung injury score obtained from 5 pathophysiological characteristics based on the histological images. **C** Alveolar neutrophils score. **D** Interstitial neutrophils score. **E** Hyaline membranes score. **F** Proteinaceous debris score. **G** Alveolar septal thickening score. **H** Representative flow cytometry plots showing the frequency of neutrophils(Ly6G+), in lung tissue. **I** Pooled flow cytometry data. Three independent experiments were performed thrice. *NS* not significant; *P* ≤ 0.05 were considered statistically significant. *P < 0.05; **P < 0.01; ***P < 0.001; ****P < 0.0001

In addition, IL-36R knockdown significantly increased pathological scores in lungs (Fig. 4A–G) 3 days after CLP surgery.

Neutrophil recruitment to the inflamed lung was increased in IL-36R−/− mice, as analyzed by FACS (Fig. 4H, I).

### IL-36R deficiency in nonhematopoietic cells served as the major contributor to the exacerbation of sepsis

**Fig. 5:**
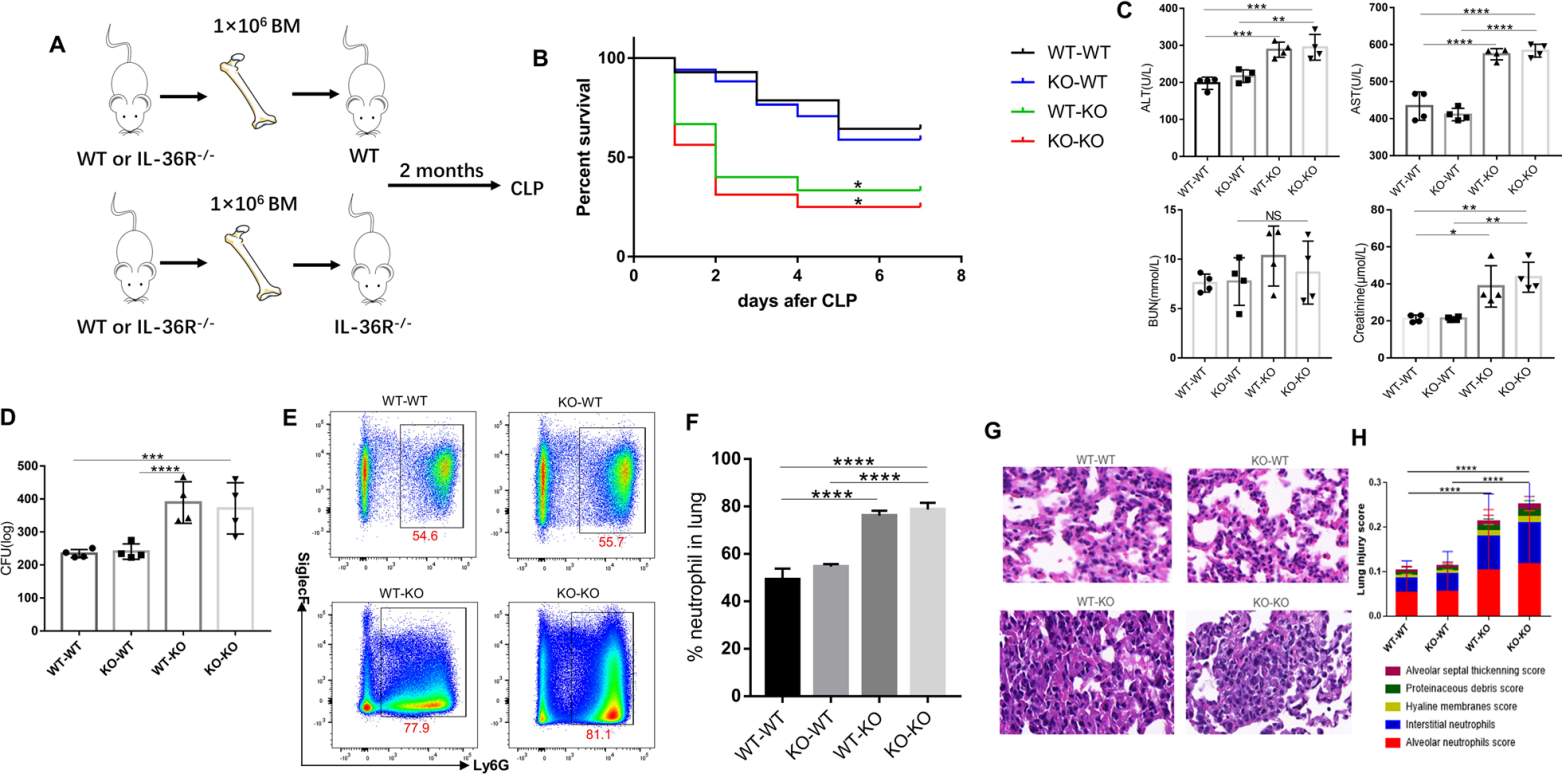
IL-36R deficiency in nonhematopoietic cells accounted for the enhanced severity to CLP-induced sepsis. **A** Scheme of BM chimeric mice construction and experimental design. **B** Survival curves of mice from the four groups of BM chimeric mice treated with CLP (n = 14 to 16 mice per group) were recorded over a 7-day period. Comparison between groups was done by Kaplan–Meier analysis followed by log–rank tests. **C** Serological markers of organ injury including alanine aminotransferase (ALT), aspartate aminotransferase (AST), blood urea nitrogen (BUN), and creatinine in CLP-induced BM chimeric mice at 3 days after CLP. Statistical difference was denoted by the horizontal bracket (Mann–Whitney U test). **D** Lung homogenates from CLP-induced BM chimeric mice were cultured on blood agar plates, and the number of bacterial colonies was counted as colony-forming unit (CFU). Statistical difference was denoted by the horizontal bracket (Mann–Whitney U test). **E** Representative flow cytometry plots showing the frequency of neutrophils(Ly6G+), in lung tissue from CLP-induced BM chimeric mice. **F** Pooled flow cytometry data. **G** The histological images of H&E stained lung sections from CLP-induced BM chimeric mice; the scale Bar = 100 μm. **H** Total lung injury score obtained from 5 pathophysiological characteristics based on the histological images. Three independent experiments were performed thrice. Statistical difference was denoted by the horizontal bracket (Mann–Whitney U test). Data are means ± SD and error bars represent SD. *P* ≤ 0.05 were considered statistically significant. *P < 0.05; **P < 0.01; ***P < 0.001; ****P < 0.0001

To assess the relative contribution of hematopoietic and nonhematopoietic cells to the sepsis exacerbation in IL-36R^−/−^ mice, we used reciprocal bone marrow (BM) transplantation and generated the following chimeric mice: WT → WT, IL-36R^−/−^ → WT, WT → IL-36R^−/−^, and IL-36R^−/−^ → IL-36R^−/−^ (Fig. 5A). At 8 week after BM transplantation, mice were subjected to CLP. During a 7-day experimental period, we found that mice with IL-36R deficiency in the nonhematopoietic compartment (WT → IL-36R^−/−^ and IL-36R^−/−^ → IL-36R^−/−^) suffered from significantly higher mortality rates (Fig. 5B). Serum concentrations of ALT and AST, markers for hepatocellular injury, and creatinine, a marker for renal failure, were significantly increased among IL-36R^−/−^ deficiency in the nonhematopoietic compartment mice at 3 days after CLP (Fig. 5C). Lung bacterial CFU levels were significantly increased in the mice with IL-36R deficiency in the nonhematopoietic compartment (WT → IL-36R^−/−^ and IL-36R^−/−^  → IL-36R^−/−^) (Fig. 5D). Neutrophils were also significantly enhanced in lung tissues resulting from IL-36R deficiency in nonhematopoietic cells (WT → IL-36R^−/−^ and IL-36R^−/−^ → IL-36R^−/−^) at 3 d after CLP (Fig. 5E, F). Histologically, mice with IL-36R^−/−^ nonhematopoietic cells (WT → IL-36R^−/−^ and IL-36R^−/−^ → IL-36R^−/−^) displayed extensive destruction of lung architecture and higher injury scores in response to CLP (Fig. 5G, H). Thus, these data suggested that IL-36R deficiency in nonhematopoietic cells, but not in BM-derived cells, exacerbated sepsis lethality and lung injury processes.

**Fig. 6:**
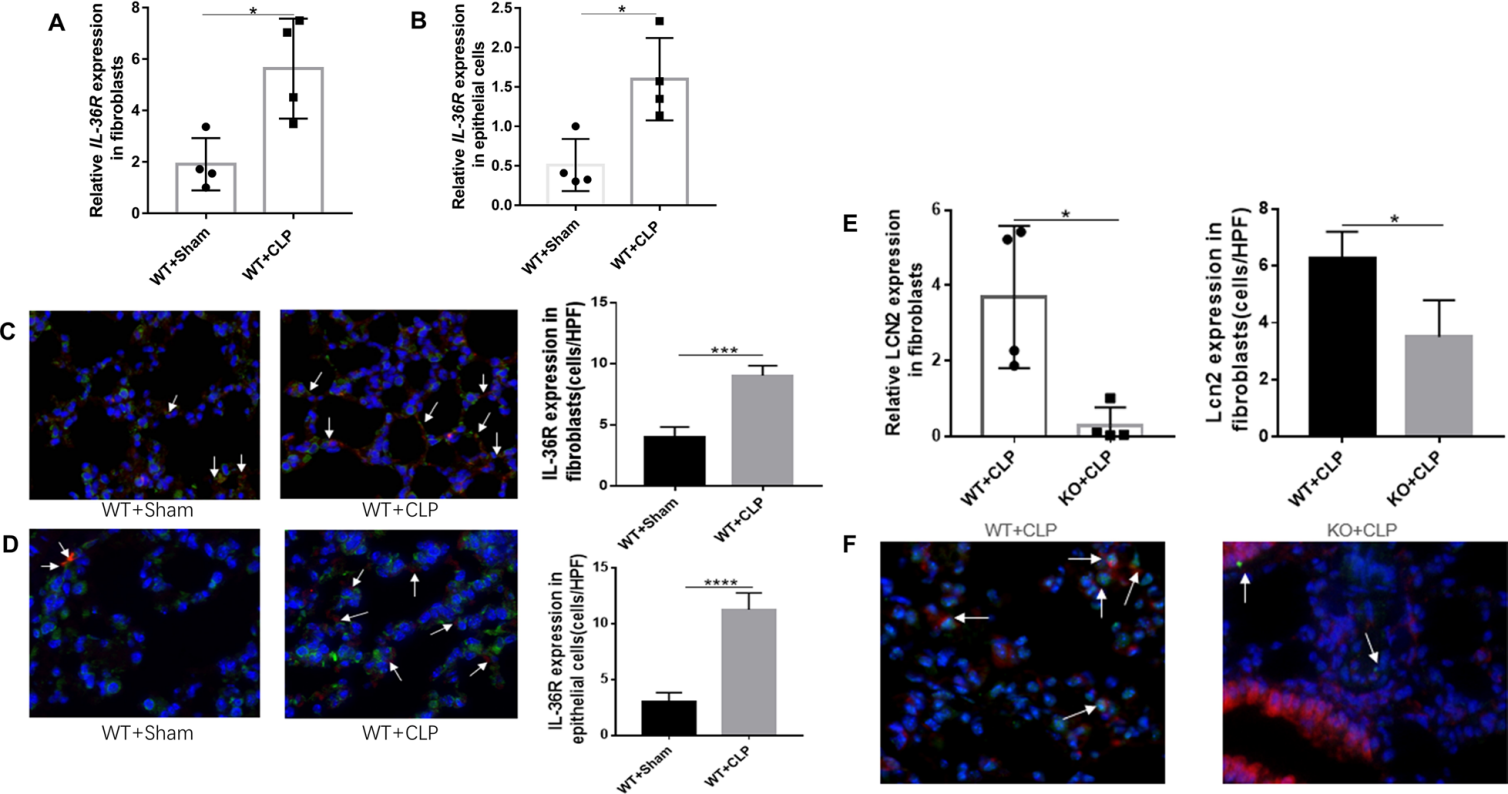
Interleukin (IL)-36R are increased in lung fibroblasts and epithelial cells. **A** Fibroblasts were purified from lung of wild type mice after CLP 3 days. Gene expression for interleukin (IL)-36R was analyzed by quantitative PCR. **B** Epithelial cells were purified from lung of wild type mice after CLP 3 days. Gene expression for interleukin (IL)-36R was analyzed by quantitative PCR. **C** Sections from lung fibroblasts in CLP-induced mice were analyzed for IL-36R by immunofluorescence (IF). Arrows indicate IL-36R expression. **D** Sections from lung epithelial cells in CLP-induced mice were analyzed for IL-36R by immunofluorescence (IF). Arrows indicate IL-36R expression. **E** Fibroblasts were purified from lung of wild type and IL-36R knock out mice after CLP 3 days. Gene expression for lipocalin 2 (LCN2) was analyzed by quantitative PCR. **F** Sections from lung fibroblasts in CLP-induced wild type and IL-36R knock out mice were analyzed for LCN2 by immunofluorescence (IF). Arrows indicate LCN2 expression. Three independent experiments were performed thrice. Statistical difference was denoted by the horizontal bracket (Mann–Whitney U test). Data are means ± SD and error bars represent SD. *P* ≤ 0.05 were considered statistically significant. *P < 0.05; **P < 0.01; ***P < 0.001; ****P < 0.0001

To identify target cell types deficient in IL-36R, we first assessed the type of each lung resident cell following sepsis, particularly the level of IL-36R expression in non-hematopoietic cells. By conducting qRT-PCR analysis for sorted lung cells, we definitely did not find any increase in IL-36R expression in neutrophils, macrophages, dendritic cells, or other hematopoietic cells after sepsis(Additional file 7: Fig. S7A-F). There is also no difference in IL-36R expression on endothelial cells (Additional file 7: Fig. S7G). We found that IL-36R expression was significantly elevated on fibroblasts and epithelial cells following sepsis, which are considered critical non-hematopoietic cells involved in the pathogenesis of septic lung injury (Fig. 6A, B). We could also observed that IL-36R is strongly expressed in lung fibroblasts and epithelial cells with sepsis as detected by immunofluorescence(Fig. 6C, D). The proportion of lung fibroblasts between KO and WT mice were not different after sepsis (Additional file 8: Fig. S8A and B). By mining a recently published lung single-cell RNA sequencing dataset on CLP-induced sepsis (GSE207651), we found that antimicrobial protein lipocalin2 (LCN2) expression was significantly elevated on fibroblasts following sepsis (Additional file 13: Table S4). Moreover, there was a significant correlation between IL-36R and LCN2 according to the published databases (Additional file 8: Fig. S8C). After sepsis, LCN2 was predominantly decreased in lung fibroblasts among KO mice (Fig. 6E) and the same results as detected by immunofluorescence (Fig. 6F).

**Fig. 7:**
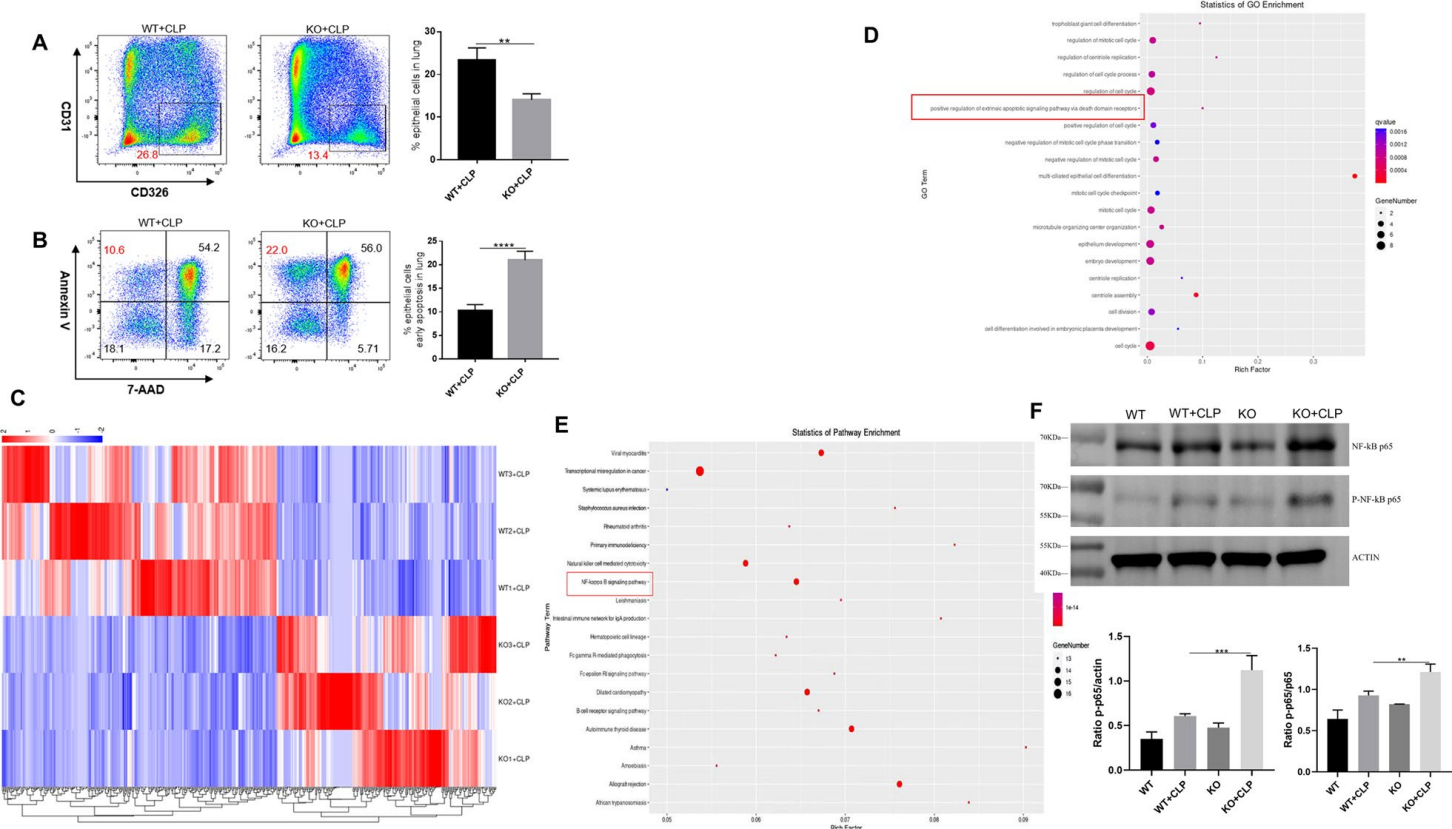
Interleukin (IL)-36R signaling promotes epithelial apoptosis via NF-κB pathway. **A** Representative flow cytometry plots showing the frequency of epithelial cells (CD326^+^), in lung tissue from CLP-induced wild type and IL-36R knock out mice. Pooled flow cytometry data of the frequency of epithelial cells. **B** Representative flow cytometry plots showing the frequency of epithelial cells apoptosis in lung tissue from CLP-induced wild type and IL-36R knock out mice. Pooled flow cytometry data of the frequency of epithelial cells apoptosis. **C** Heat map of lung epithelial cells RNA-seq (n = 3) from wild type (WT) and IL-36R knock out (KO) mice after CLP 3 days. The fluorescence intensity of differentially expressed RNAs (≥ twofold) is illustrated from high (red) to low (blue). **D** Gene ontology (GO) analysis on differentially expressed RNAs. **E** Kyoto Encyclopedia of Genes and Genomes (KEGG) analysis on differentially expressed RNAs; the 20 most enriched pathways related to signaling transduction are shown. **F** Representative western blot of P65 and p-P65 in the epithelial cells. Protein quantification of P65, p-P65 in the epithelial cells. Three independent experiments were performed thrice. *P* ≤ 0.05 were considered statistically significant. *P < 0.05; **P < 0.01; ***P < 0.001; ****P < 0.0001

To assess the role of epithelial cell in this process, we also examined the propotion of lung epithelial cells between KO and WT mice. We found that the proportion of lung epithelial cells significantly decreased among KO mice after sepsis (Fig. 7A). To determine whether IL-36R deletion could functionally influence the lung epithelial cells death, we analysed the death of epithelial cells by 7-AAD and Annexin V. We detected a marked induction of epithelial cells that stained positive for Annexin V and negative for 7-AAD. These results indicating that IL-36R deletion could promote the early apoptosis of the lung epithelial cells (Fig. 7B). In subsequent studies, we wanted to perform a comprehensive analysis of the molecular mechanisms induced by IL-36R signalling in lung epithelial cells. Therefore, we sorted epithelial cells from the lung of WT and KO mice in the presence CLP for 3 days. Subsequently, RNA was purified from both groups and whole genome expression profiling was performed by RNA-seq. RNA-seq data have been deposited in the public database GEO with accession number GSE240924. Hierarchical clustering of samples showed high similarities in overall transcription patterns upon IL-36R knockout (Fig. 7C). Gene ontology (GO) based functional annotation analysis with the differentially expressed and upregulated genes revealed that there was a significant enrichment for early apoptosis in lung epithelial cells with IL-36R deletion (Fig. 7D). Through KEGG analysis, the nuclear factor κB (NF-κB) pathway on lung epithelial cells was activated with IL-36R deletion (Fig. 7E). In summary, the RNA-seq studies with primary lung epithelial cells demonstrated that such cells are broadly modulated by IL-36R activation resulting in characteristic gene expression patterns with NF-κB. The results of WB are consistent with those of RNA-seq (Fig. 7F and Additional file 9: Fig. S9).

## Discussion

Sepsis is characterized by life-threatening organ dysfunction caused by systemic inflammation and immune dysregulation in the host [23, 24]. Cytokines play an important role in sepsis pathogenesis [20, 25, 26]. IL-36, as a member of the IL-1 cytokine family, plays an important role in the coordination of innate and adaptive immunity [11]. This study first assessed the serum level of IL-36 in sepsis patients, and three main results were obtained. First, circulating IL-36α, IL-36β and IL-36γ levels of sepsis patients on the day of admission were significantly higher than those of ICU controls and healthy individuals. Second, the serum IL-36 family member's levels were related to the severity of sepsis, including CRP, PCT, and SOFA scores. Third, a high serum IL-36 level on the day of admission was associated with sepsis diagnosis and 28-day mortality in septic patients.

Previously, others have demonstrated increased levels of IL-36 family members in human patients with various inflammatory diseases. In patients with IBD (inflammatory bowel disease), IL-36α and IL-36γ are strongly expressed in mucosal biopsies from the colon of patients with IBD and the expression of IL-36α and IL-36γ was associated with the degree of inflammation in IBD patients [27]. In ARDS (acute respiratory distress syndrome), the plasma and BALF concentrations of IL-36γ were higher in patients with P. aeruginosa-induced ARDS than those of healthy controls [14]. The current study further extends these findings, clarifies the increase of serum IL-36 family members in a cohort of sepsis patients, and refines information on the association between serum IL-36 levels and sepsis disease severity. Importantly, on the day of admission, significantly higher serum IL-36 levels were observed in septic survivors than in septic nonsurvivors. Another anti-inflammatory cytokine in IL-1 family, IL-1Ra, also significantly elevated in sepsis [28].

Although the AUC of IL-36α and IL-36β were lower than that of the SOFA score in predicting 28-day mortality in septic patients, they were superior to PCT for predicting 28-day mortality in septic patients. IL-36γ was higher than that of the SOFA score in predicting 28-day mortality in septic patients. In general, these findings not only suggest that IL-36 may be a potential biomarker of sepsis reflecting disease severity and mortality, but also encourage further investigation on its role in the pathogenesis of sepsis.

Here, we have investigated the role of IL-36 in the progression of polymicrobial sepsis by establishing a clinically relevant murine model of sepsis induced by CLP. Since the mouse genome contains homologs of the IL-36R gene, we used IL-36R knockout mice to detect the role of the IL-36R signal during sepsis.

As mentioned earlier, IL-36α and IL-36β contribute to the improvement of the host immune response [29, 30], and we found that IL-36R deletion aggravated CLP-induced sepsis mortality. The increased mortality was associated with damaged bacterial clearance and multiple organ injuries, especially the lung. Unexpectedly, the results from reciprocal BM transplantation experiments indicated that IL-36R deficiency in nonhematopoietic cells served as the major contributor to the aggravation of sepsis. Among the resident nonhematopoietic compartments in the lung, lung fibroblasts and epithelial cells play important roles in lung injury pathogenesis [31, 32]. We find that IL-36R expression was significantly elevated on fibroblasts and epithelial cells following sepsis. IL-36R deficiency in the nonhematopoietic increased the bacterial burden. In the meantime, the accumulation of neutrophils injured the lung. Excessive neutrophils injured the host and low LCN2 level increased the bacterial burden [33, 34].

LCN2 was additionally identified as a target of IL-36R signalling in lung fibroblasts. LCN2, as a bacteriostatic factor, blocks iron acquisition by bacterial siderophores, thereby inhibiting bacterial growth [35]. One study showed higher mortality in LCN2 knockout mice following systemic administration of *E. coli* [36]. In contrast, upregulated LCN2 mediated the bacteriostatic effect of *E. coli* in WT mice, protecting mice from death caused by sepsis [37]. Interestingly, we could also observe a devastating effect by LCN2 lower expression in IL-36R^−/−^ mice in sepsis suggesting that LCN2 is one of the key factors driving IL-36-dependent lung injury.

Sepsis could trigger the inflammatory cascade, inducing apoptosis of lung epithelial cells [38]. Current evidence suggests that disruption of the epithelial barrier due to the epithelial cells apoptosis may be one of the core detrimental of septic acute lung injury [39, 40]. Epithelial-barrier integrity damage allows the accumulation of inflamed cells and the leakage of plasma proteins into alveolar space, preventing the normal gas exchange [41]. In our study, epithelial cells apoptosis may caused the accumulation of neutrophils. As a transcription factor, NF-κB plays a pivotal role in control of apoptosis, and cell survival, and is associated with the sepsis-induced lung injury [42]. The results of RNA-seq and WB indicating that epithelial cells apoptosis on sepsis induced acute lung injury was mediated through the NF-κB pathway.

Despite the findings, this study has some limitations. First, we examined only a limited number of sepsis patients, and the relationships between IL-36 family members' levels and disease severity or mortality in sepsis patients should be validated in a larger ideally multicentric study. Second, although healthy controls and non-sepsis critical illness subjects were included in this study, other groups of patient controls should be better included. Third, the kinetics of IL-36 secretion, the cellular source of IL-36, and the factors regulating IL-36R function in human sepsis remain unknown.

## Conclusions

Collectively, our study first showed the role of IL-36 family members during sepsis in both human and murine models. Although our findings do not exclude additional direct and indirect effects of IL-36R ligands on immune cells, the results suggest that IL-36R signaling in fibroblasts and epithelial cells is an important pathway for the maintenance of lung homeostasis and the pathogenesis of sepsis. Thus, modulation of the IL-36R pathway emerges as a potential therapeutic strategy for lung injury in sepsis.

### Supplementary Information

**Additional file 1**. **Figure S1** IL-36R and IL-36 subtypes mRNA expression in blood samples of sepsis patients (n = 18) compared with normal controls (n = 34) based on the reanalysis of a published dataset (GSE69063). Data in A, B, C are representative of 0, 1, 3 h post arrival. Data are shown as mean ± SEM; NS, not significant; *P < 0.05, **P < 0.01 by two-tailed Student’s t test.

**Additional file 2**. **Figure S2 Receiver operating characteristic curve (ROC) of Interleukin (IL)-36 subtypes for diagnosis of sepsis.** Areas under the ROC curve for IL-36α, 0.797 (*p* < 0.001). Areas under the ROC curve for IL-36β, 0.681 (*p* = 0.005). Areas under the ROC curve for IL-36γ, 0.863 (*p* < 0.001).

**Additional file 3**. **Figure S3 Receiver operating characteristic curve (ROC) of interleukin (IL)-36 subtypes for predicting 28-day mortality in septic patients.** ROC curve of interleukin (IL)-36 subtypes, SOFA score, PCT at admission for predicting 28-day mortality in septic patients. Area under the ROC curve, 0.730 (*p* = 0.004) for IL-36α, 0.706 (*p* = 0.011) for IL-36β, 0.756 (*p* = 0.003) for IL-36γ, 0.738 (*p* = 0.011) for SOFA score, and 0.649 (*p* = 0.153) for PCT.

**Additional file 4**. **Figure S4** Systemic interleukin (IL)-36 subtypes levels in mice with sepsis. Lung were removed at 3 and 7 days after CLP, blood was obtained by cardiac puncture. Samples were assayed for IL-36α, IL-36β, and IL-36γ content by specific sandwich enzyme-linked immunosorbent assay (ELISA). Three independent experiments were performed thrice. *P < 0.05; **P < 0.01; ***P < 0.001(vs sham controls; Mann–Whitney U test).

**Additional file 5**. **Figure S5** Systemic interleukin (IL)-1Ra levels in mice and patients with sepsis. Blood were obtained at 3 and 7 days after CLP. Samples were assayed for IL-1Ra content by specific sandwich enzyme-linked immunosorbent assay (ELISA). *P < 0.05; **P < 0.01; ***P < 0.001(vs sham controls; Mann–Whitney U test).

**Additional file 6**. **Figure S6.** Interleukin (IL)- 36R is highly expressed in the lung. (A) IL-36R expression in different tissues was plotted by analyzing a published RNA-Seq dataset (GSE179554). (B) The expression of IL-36R ligands was analysed in heart, kidney, liver, and lung tissues by quantitative PCR. Three independent experiments were performed thrice.

**Additional file 7**. **Figure S7.** Interleukin (IL)- 36R is expressed in hematopoietic cells after sepsis. (A) The expression of IL-36R ligands was analysed in neutrophils by quantitative PCR at 3 days after CLP. (B) The expression of IL-36R ligands was analysed in macrophages by quantitative PCR at 3 days after CLP. (C) The expression of IL-36R ligands was analysed in T cells by quantitative PCR at 3 days after CLP. (D) The expression of IL-36R ligands was analysed in B cells by quantitative PCR at 3 days after CLP. (E) The expression of IL-36R ligands was analysed in natural killer cells by quantitative PCR at 3 days after CLP. (F) The expression of IL-36R ligands was analysed in dendritic cells by quantitative PCR at 3 days after CLP. (G) The expression of IL-36R ligands was analysed in endothelial cells by quantitative PCR at 3 days after CLP. Three independent experiments were performed thrice.

**Additional file 8**. **Figure S8.** The changes of lung fibroblasts after sepsis. (A) Flow cytometry of lung fibroblasts after sepsis at 3 days. (B) The proportion of fibroblasts in lung tissues were quantified. (C) Correlation of IL-36R levels with LCN2 levels in the patients with sepsis. Three independent experiments were performed thrice.

**Additional file 9**. **Figure  S9.** Uncropped gel and blot images have been provided as supplementary files.

**Additional file 10**. **Table S1.** Characteristics of septic patients, ICU controls, and healthy controls.

**Additional file 11**. **Table S2.** Primers used for qRT-PCR.

**Additional file 12**. **Table S3.** Antibody list for flow cytometry.

**Additional file 13**. **Table S4.** Expression of the top 5 differential genes on fibroblasts (GSE207651).

## Data Availability

The datasets generated and analyzed during the current study are available from the first author on reasonable request.
