# Supplementary figures and images for: Interleukin-36 is overexpressed in human sepsis and IL-36 receptor deletion aggravates lung injury and mortality through epithelial cells and fibroblasts in experimental murine sepsis
Source: Crit Care. 2023 Dec 13;27:490. doi: 10.1186/s13054-023-04777-z (PMC10717293; doi:10.1186/s13054-023-04777-z)

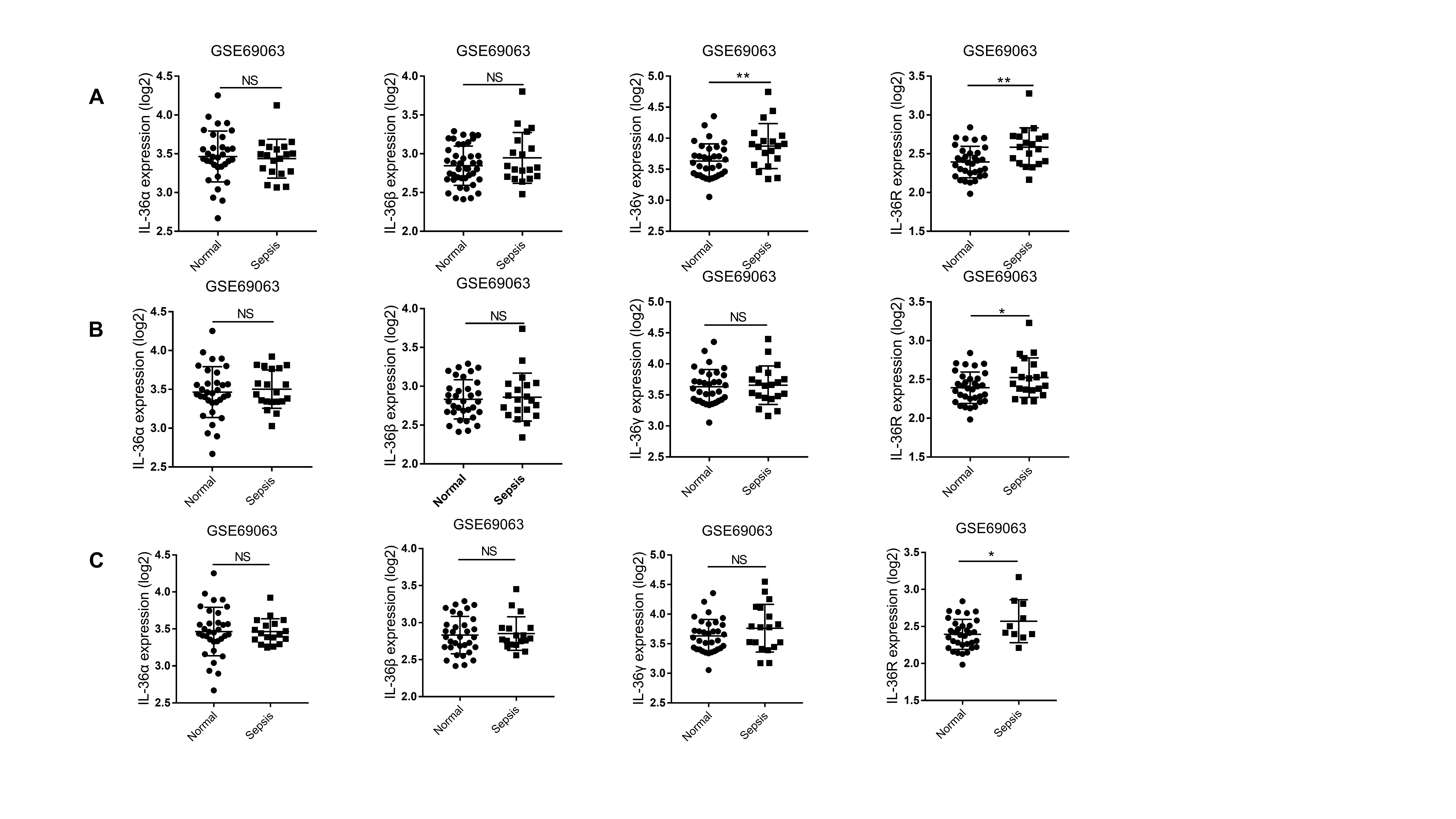

Supplement: Supplementary file 1 — Additional file 1. Figure S1 IL-36R and IL-36 subtypes mRNA expression in blood samples of sepsis patients (n = 18) compared with normal controls (n = 34) based on the reanalysis of a published dataset (GSE69063). Data in A, B, C are representative of 0, 1, 3 h post arrival. Data are shown as mean ± SEM; NS, not significant; *P < 0.05, **P < 0.01 by two-tailed Student’s t test. [file 13054_2023_4777_MOESM1_ESM.jpg]

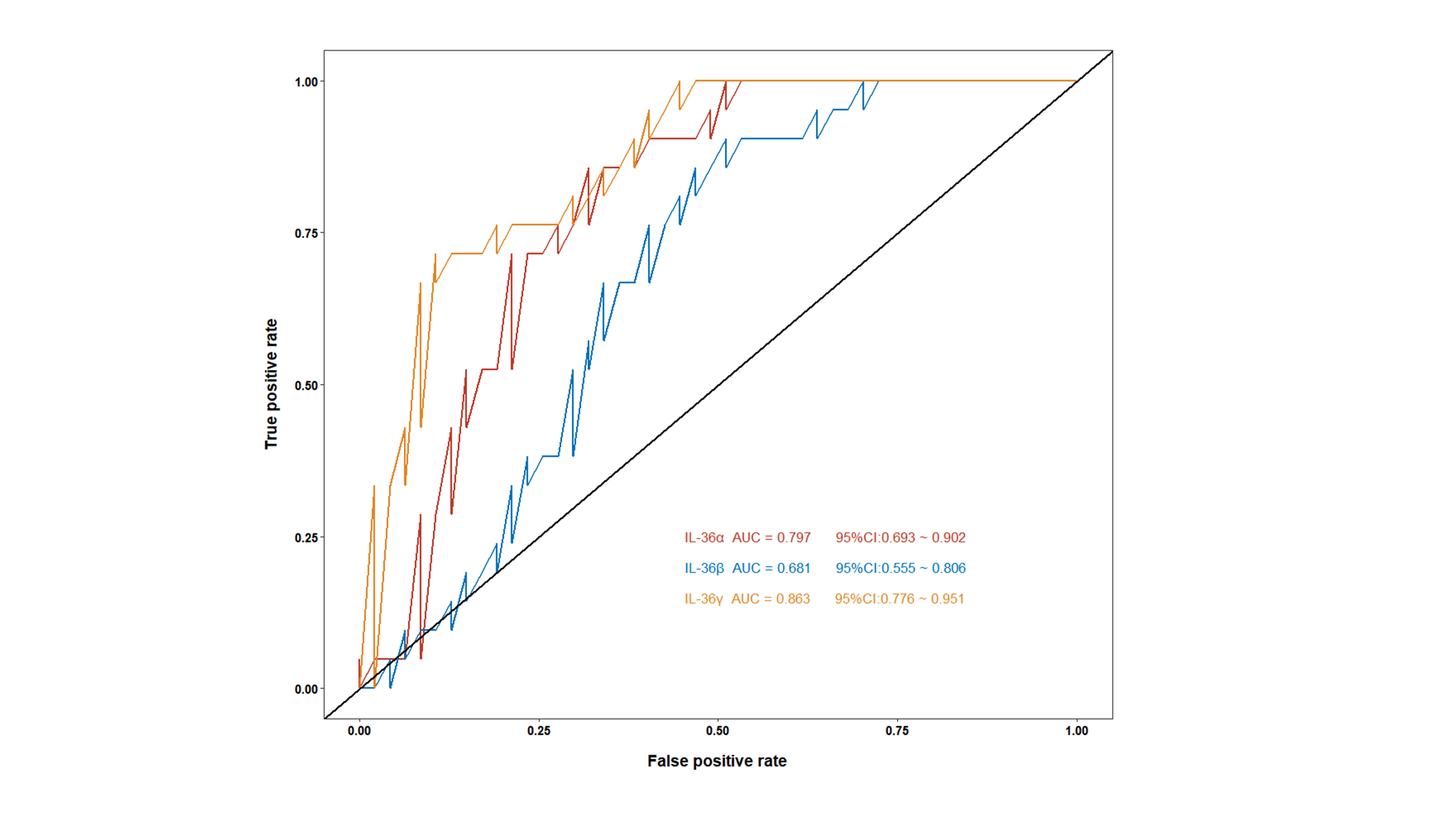

Supplement: Supplementary file 2 — Additional file 2. Figure S2 Receiver operating characteristic curve (ROC) of Interleukin (IL)-36 subtypes for diagnosis of sepsis. Areas under the ROC curve for IL-36α, 0.797 (p < 0.001). Areas under the ROC curve for IL-36β, 0.681 (p = 0.005). Areas under the ROC curve for IL-36γ, 0.863 (p < 0.001). [file 13054_2023_4777_MOESM2_ESM.jpg]

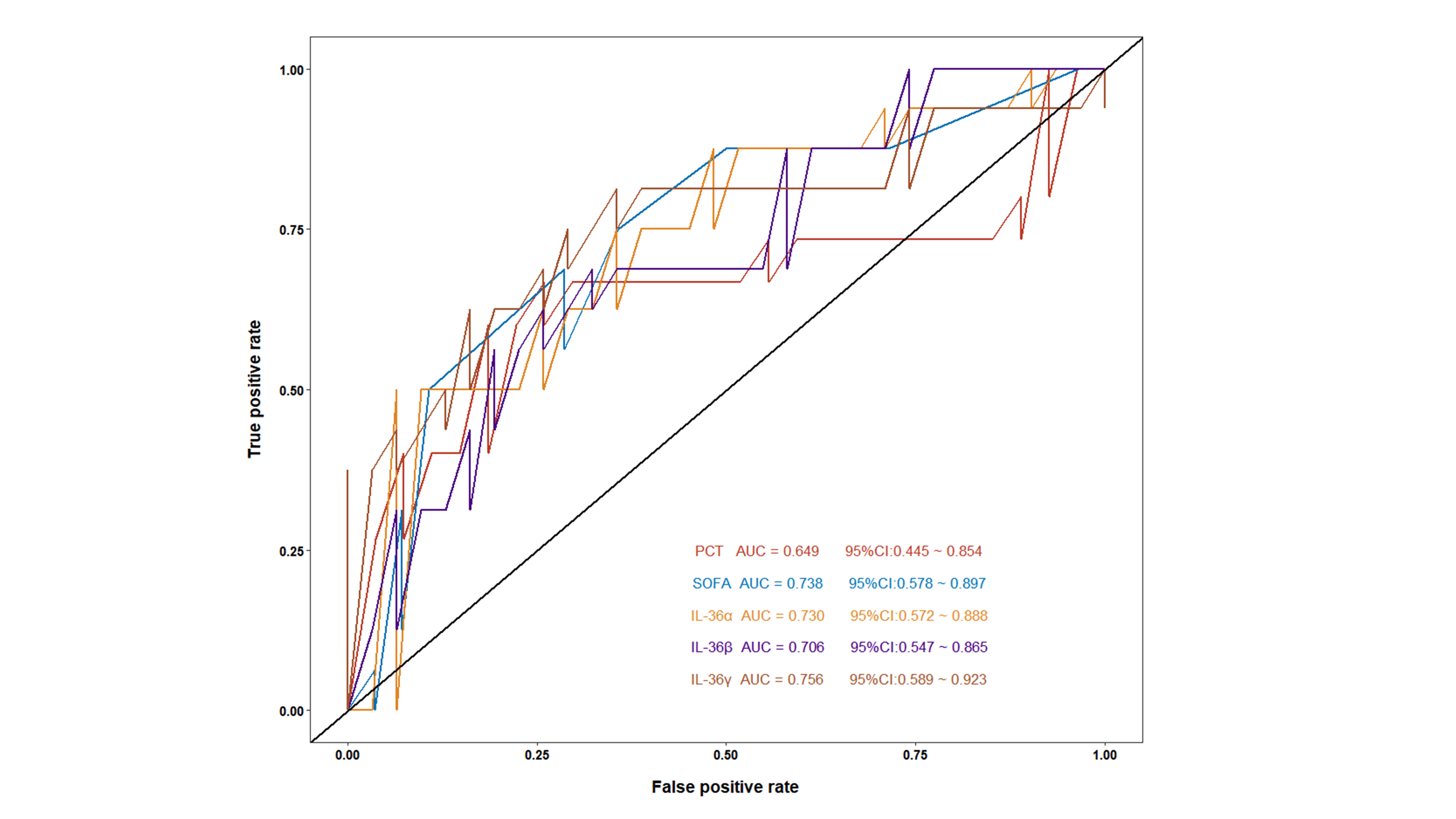

Supplement: Supplementary file 3 — Additional file 3. Figure S3 Receiver operating characteristic curve (ROC) of interleukin (IL)-36 subtypes for predicting 28-day mortality in septic patients. ROC curve of interleukin (IL)-36 subtypes, SOFA score, PCT at admission for predicting 28-day mortality in septic patients. Area under the ROC curve, 0.730 (p = 0.004) for IL-36α, 0.706 (p = 0.011) for IL-36β, 0.756 (p = 0.003) for IL-36γ, 0.738 (p = 0.011) for SOFA score, and 0.649 (p = 0.153) for PCT. [file 13054_2023_4777_MOESM3_ESM.jpg]

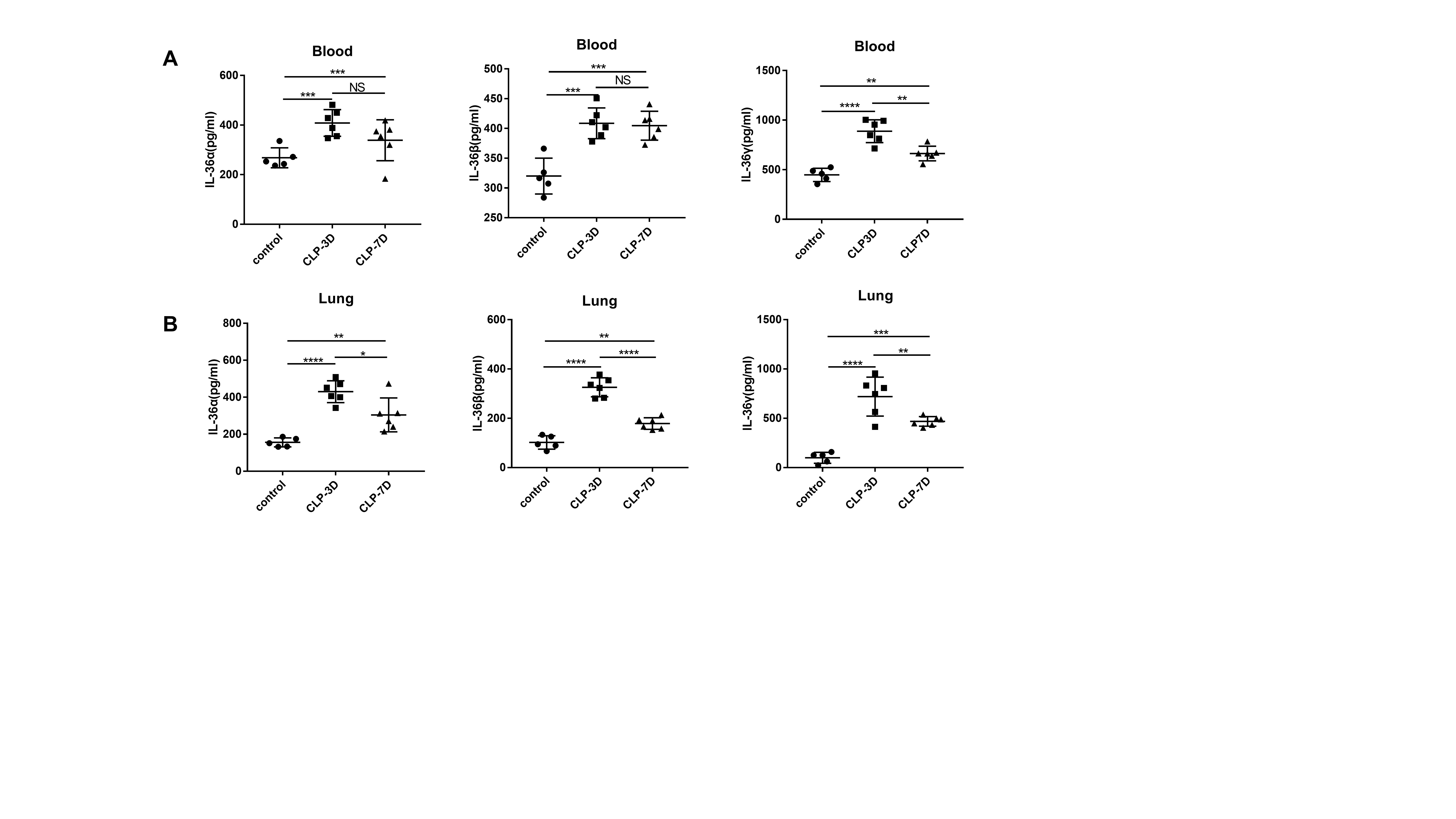

Supplement: Supplementary file 4 — Additional file 4. Figure S4 Systemic interleukin (IL)-36 subtypes levels in mice with sepsis. Lung were removed at 3 and 7 days after CLP, blood was obtained by cardiac puncture. Samples were assayed for IL-36α, IL-36β, and IL-36γ content by specific sandwich enzyme-linked immunosorbent assay (ELISA). Three independent experiments were performed thrice. *P < 0.05; **P < 0.01; ***P < 0.001(vs sham controls; Mann–Whitney U test). [file 13054_2023_4777_MOESM4_ESM.jpg]

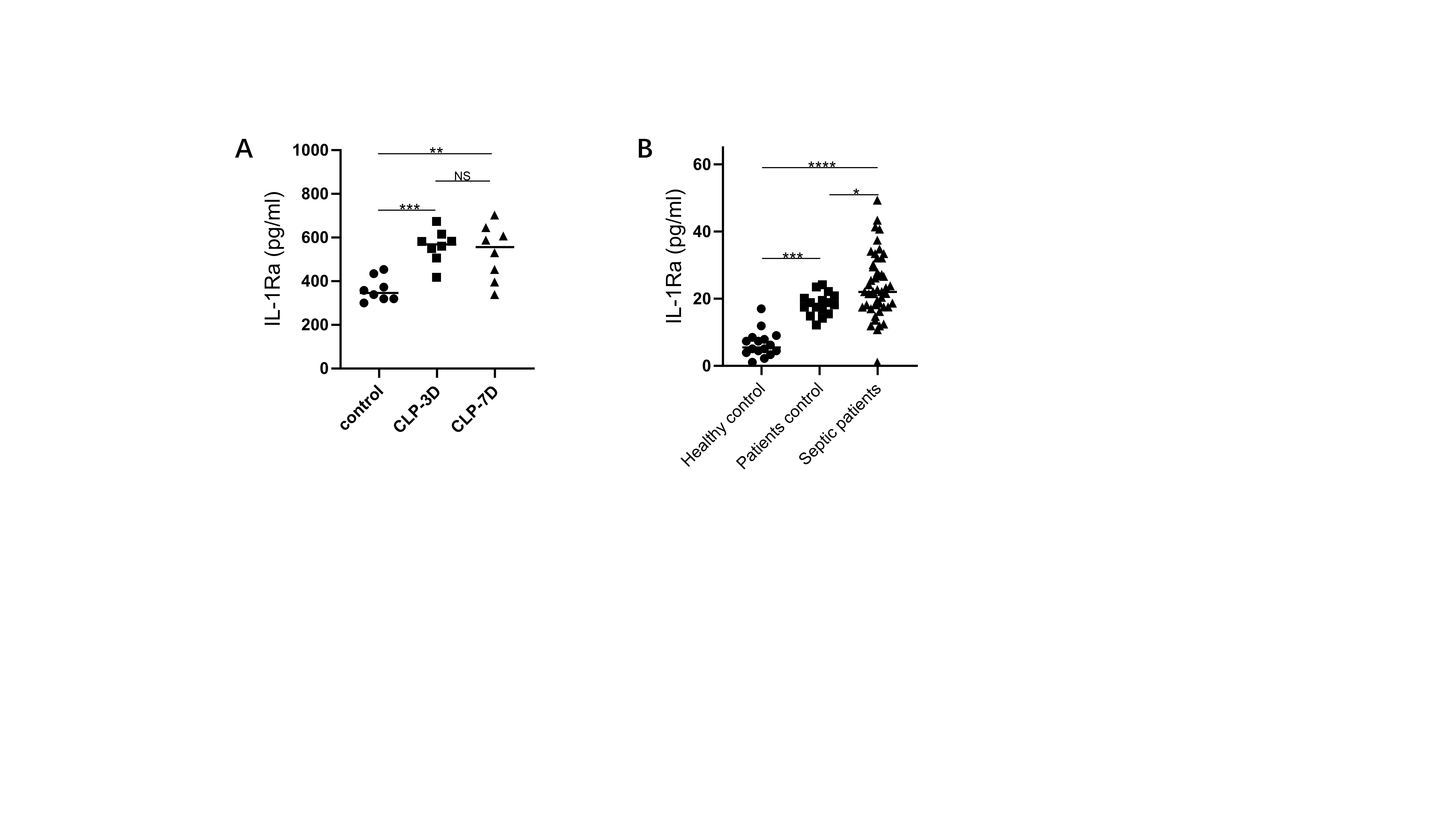

Supplement: Supplementary file 5 — Additional file 5. Figure S5 Systemic interleukin (IL)-1Ra levels in mice and patients with sepsis. Blood were obtained at 3 and 7 days after CLP. Samples were assayed for IL-1Ra content by specific sandwich enzyme-linked immunosorbent assay (ELISA). *P < 0.05; **P < 0.01; ***P < 0.001(vs sham controls; Mann–Whitney U test). [file 13054_2023_4777_MOESM5_ESM.jpg]

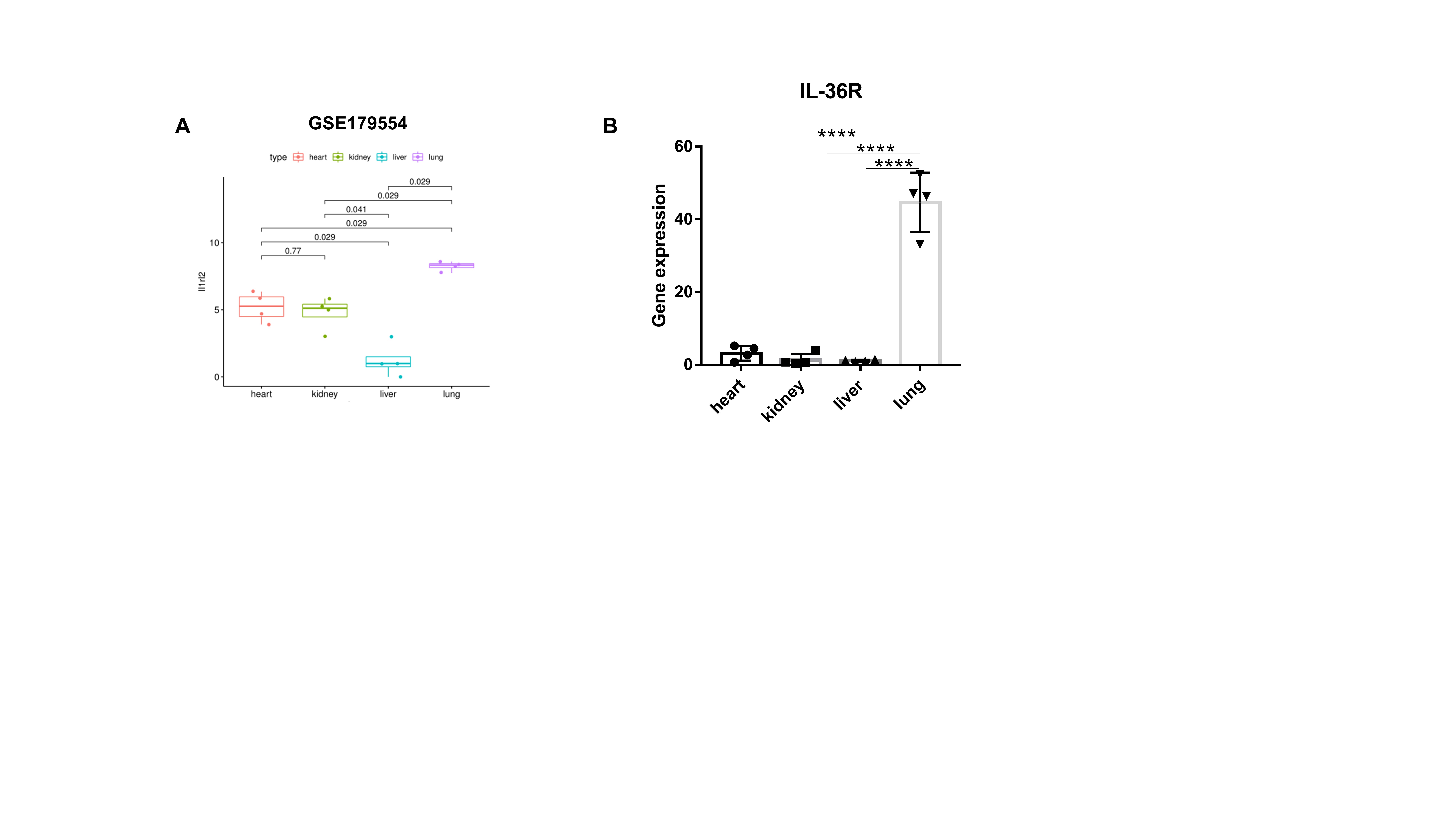

Supplement: Supplementary file 6 — Additional file 6. Figure S6. Interleukin (IL)- 36R is highly expressed in the lung. (A) IL-36R expression in different tissues was plotted by analyzing a published RNA-Seq dataset (GSE179554). (B) The expression of IL-36R ligands was analysed in heart, kidney, liver, and lung tissues by quantitative PCR. Three independent experiments were performed thrice. [file 13054_2023_4777_MOESM6_ESM.jpg]

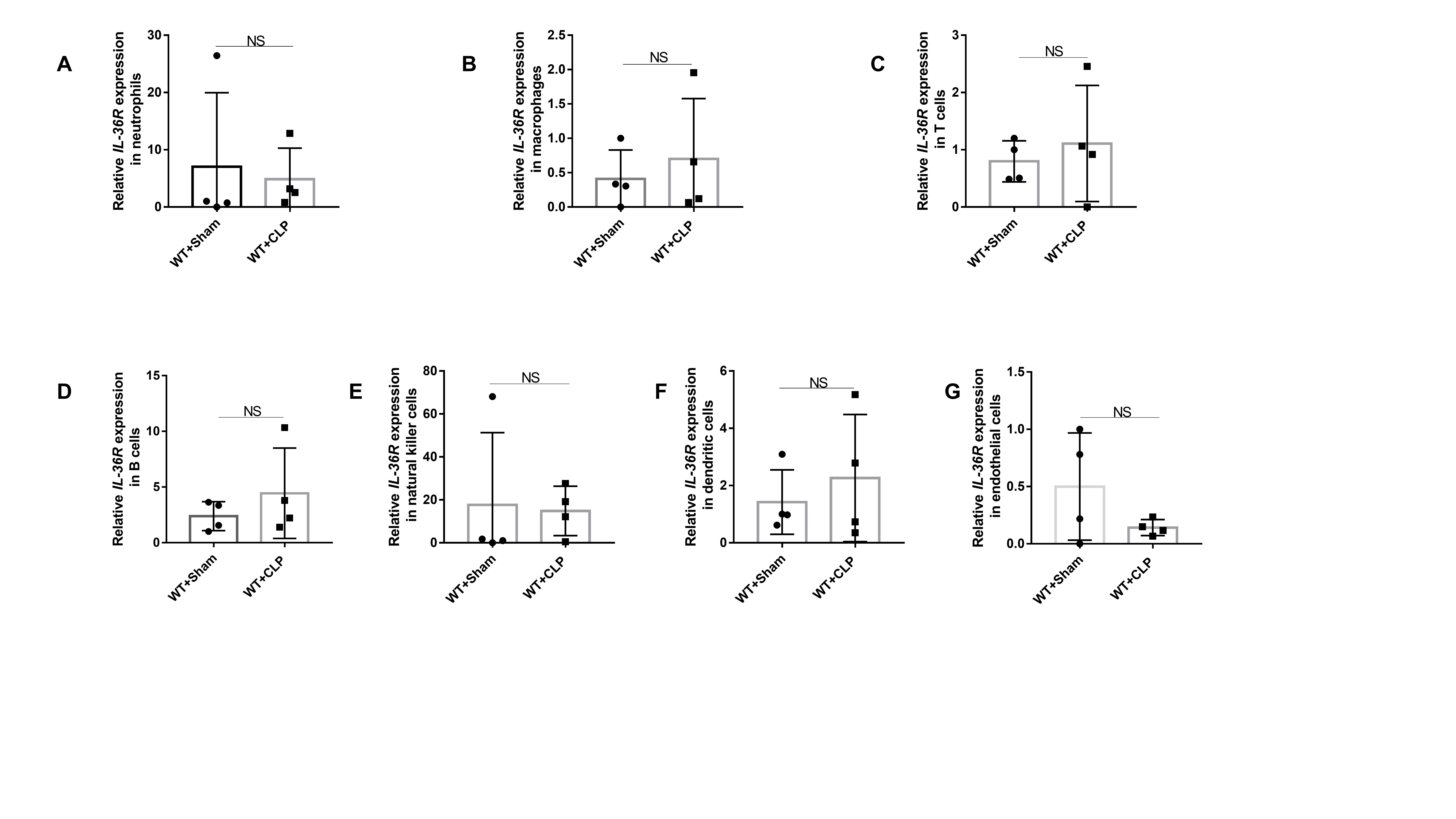

Supplement: Supplementary file 7 — Additional file 7. Figure S7. Interleukin (IL)- 36R is expressed in hematopoietic cells after sepsis. (A) The expression of IL-36R ligands was analysed in neutrophils by quantitative PCR at 3 days after CLP. (B) The expression of IL-36R ligands was analysed in macrophages by quantitative PCR at 3 days after CLP. (C) The expression of IL-36R ligands was analysed in T cells by quantitative PCR at 3 days after CLP. (D) The expression of IL-36R ligands was analysed in B cells by quantitative PCR at 3 days after CLP. (E) The expression of IL-36R ligands was analysed in natural killer cells by quantitative PCR at 3 days after CLP. (F) The expression of IL-36R ligands was analysed in dendritic cells by quantitative PCR at 3 days after CLP. (G) The expression of IL-36R ligands was analysed in endothelial cells by quantitative PCR at 3 days after CLP. Three independent experiments were performed thrice. [file 13054_2023_4777_MOESM7_ESM.jpg]

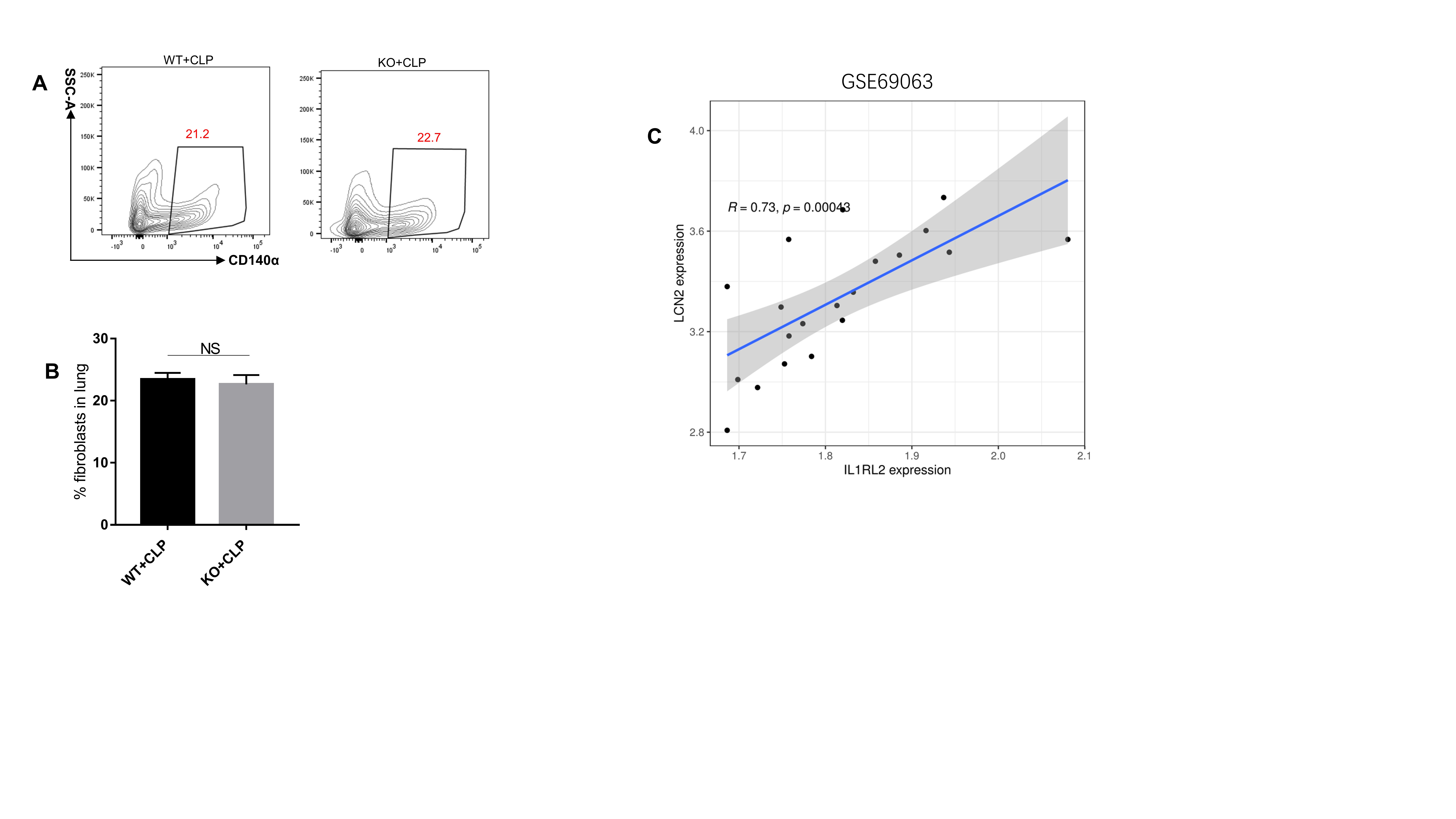

Supplement: Supplementary file 8 — Additional file 8. Figure S8. The changes of lung fibroblasts after sepsis. (A) Flow cytometry of lung fibroblasts after sepsis at 3 days. (B) The proportion of fibroblasts in lung tissues were quantified. (C) Correlation of IL-36R levels with LCN2 levels in the patients with sepsis. Three independent experiments were performed thrice. [file 13054_2023_4777_MOESM8_ESM.jpg]

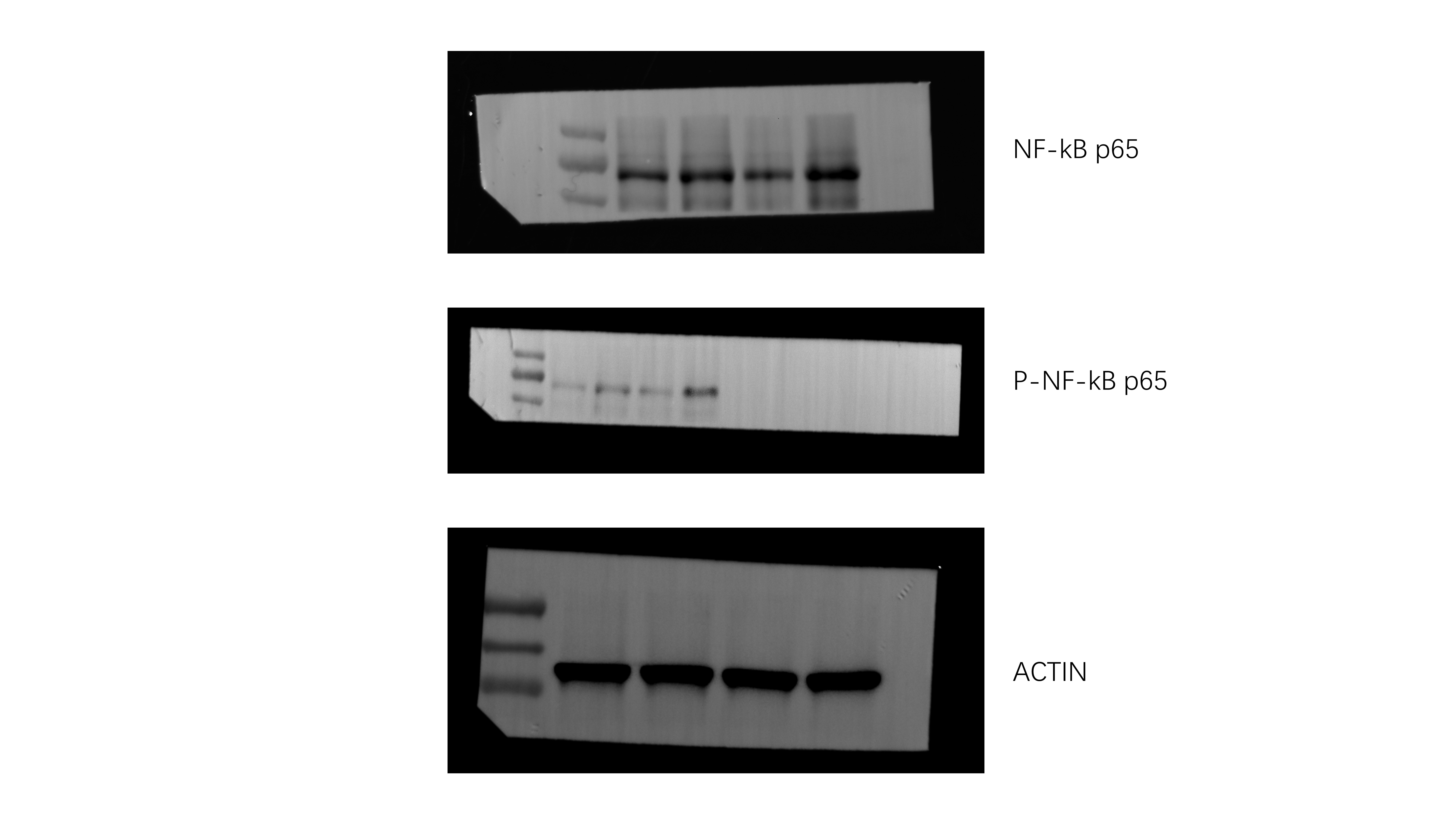

Supplement: Supplementary file 9 — Additional file 9. Figure S9. Uncropped gel and blot images have been provided as supplementary files. [file 13054_2023_4777_MOESM9_ESM.jpg]
