# Supplementary material for: Interleukin-36 is overexpressed in human sepsis and IL-36 receptor deletion aggravates lung injury and mortality through epithelial cells and fibroblasts in experimental murine sepsis
Source: Crit Care. 2023 Dec 13;27:490. doi: 10.1186/s13054-023-04777-z (PMC10717293; doi:10.1186/s13054-023-04777-z)
Supplement: Supplementary file 10 — Additional file 10. Table S1. Characteristics of septic patients, ICU controls, and healthy controls. [file 13054_2023_4777_MOESM10_ESM.docx]

Table S1 Characteristics of septic patients, ICU controls, and healthy controls

| Characteristics | Sepsis patients (n=47) | ICU controls (n=21) | Healthy controls (n=21) |
| --- | --- | --- | --- |
| Age, year | 72(69-76) | 67(61-72) | 66(63-69) |
| Female | 18 | 7 | 9 |
| T(℃) | 38.5(37-39.4) | 36.8(36.3-37.8) | NA |
| SOFA score | 7.0(3-12) | 1.0(0-2) | NA |
| APACHEⅡ | 33(28-36) | 24(20-28) | NA |
| Septic shock | 12 | 0 | 0 |
| Mechanical ventilation | 32 | 11 | 0 |
| PaO2/FiO2[mmHg(kPa)] | 261.4(189.2-354.1) | 238.6(138.1-372.3) | NA |
| BMI(kg/m^2^) | 23.8(19.1-25.6) | 24.9(22.9-26.0) | NA |
| FPG(mmol/L) | 7.9(6.7-12.8) | 9.6(6.2-11.1) | 5.3(5.0-5.9) |
| WBC(10^9/L) | 17.2(11.4-21.4) | 10.7(9.1-17.1) | 6.4(5.5-8.1) |
| NEU(%) | 89.7(83.8-92.6) | 87.2(82.0-90.5) | 55.7(50.0-57.0) |
| Mon(10^9/L) | 0.8(0.5-1.2) | 0.5(0.3-1.0) | 5.7(5-6.7) |
| PLT(10^9/L) | 194(115-275) | 199(136-280) | 229(192-278) |
| AST(U/L) | 33.3(24.0-117.5) | 31.6(20.5-65.9) | 19.1(17.0-24.8) |
| ALT(U/L) | 28(17.9-58.5) | 28.9(13.7-63) | 25.0(16.9-29.8) |
| Alb(g/L) | 32.7(28.5-36.8) | 34.1(30.3-39.8) | 44.7(43.2-46.5) |
| UA(μmol/L) | 370.9(255.6-526.1) | 499.0(298.9-576.4) | 417.5(312.3-461.1) |
| Cre(umol/L) | 101.2(64.0-180.7) | 100.8(62.2-289.2) | 81.8(69.6-90.7) |
| cTnI(ng/ml) | 0.06(0.03-1.1) | 0.4(0.03-3.5) | NA |
| NT-proBNP(pg/ml) | 1840.6(807.0-6492.5) | 1040.0(436.6-3578.9) | NA |
| CK(U/L) | 223(92-631.5) | 86.1(45.0-323.3) | NA |
| CK-MB(U/L) | 12.4(7.4-35.5) | 14.0(3.2-24.4) | NA |
| Lac(mmol/L) | 2.3(1.1-3.5) | 1.5(1.1-2.4) | NA |
| PCT, ng/ml | 13.9(2.2-25.5) | 2.5(0.0-10.0) | NA |
| CRP, mg/L | 124.0(72.9-175.0) | 12.0(4.9-19.2) | NA |
| Infection site |  |  |  |
| Respiratory | 18 | NA | NA |
| Abdominal | 10 | NA | NA |
| Urinary | 13 | NA | NA |
| Others | 7 | NA | NA |
| 28-d mortality | 16(34%) | 0(0%) | NA |

NOTE. Data are expressed as median (interquartile range) unless otherwise indicated.

ICU: intensive care unit; T: temperature; APACHE: Acute Physiology and Chronic Health Evaluation; BMI: Body Mass Index; FPG: Fasting Plasma Glucose; WBC: white blood cell; NEU: Neutrophil; Mon: Monocytes; PLT: platelet; AST: aspartate transaminase; ALT: alanine transaminase; Alb: Albumin; UA: uric acid; Cre: creatine; cTnI: Cardiac troponin I; NT-proBNP: N-Terminal pro-brain natriuretic peptide; CK: Creatine Kinase; CK-MB: creatine kinase isoenzymes; Lac: Lactic acid; PCT: procalcitonin; CRP: C-reaction protein; SOFA: sequential organ failure assessment; NA: not applicable.
