# Supplementary material for: Interleukin-36 is overexpressed in human sepsis and IL-36 receptor deletion aggravates lung injury and mortality through epithelial cells and fibroblasts in experimental murine sepsis
Source: Crit Care. 2023 Dec 13;27:490. doi: 10.1186/s13054-023-04777-z (PMC10717293; doi:10.1186/s13054-023-04777-z)
Supplement: Supplementary file 11 — Additional file 11. Table S2. Primers used for qRT-PCR. [file 13054_2023_4777_MOESM11_ESM.docx]

| Table S2. Primers used for qRT-PCR | | |
| --- | --- | --- |
| Gene | Forward Primer (5'-3') | Reverse Primer (5'-3') |
| *Gapdh* | CATCACTGCCACCCAGAAGACTG | ATGCCAGTGAGCTTCCCGTTCAG |
| *IL-36R* | TCGGGCAGTCTGAATTGTCA | GCTCCTGAAGGGCTGTACTT |
| *S100a9* | TGGTGGAAGCACAGTTGGCAAC | CAGCATCATACACTCCTCAAAGC |
| *S100a8* | CAAGGAAATCACCATGCCCTCTA | ACCATCGCAAGGAACTCCTCGA |
| *Lcn2* | ATGTCACCTCCATCCTGGTCAG | GCCACTTGCACATTGTAGCTCTG |
| *Cxcl1* | TCCAGAGCTTGAAGGTGTTGCC | AACCAAGGGAGCTTCAGGGTCA |
| *Cxcl2* | CATCCAGAGCTTGAGTGTGACG | GGCTTCAGGGTCAAGGCAAACT |
| *Gm-csf* | AACCTCCTGGATGACATGCCTG | AAATTGCCCCGTAGACCCTGCT |
