# Supplementary material for: Interleukin-36 is overexpressed in human sepsis and IL-36 receptor deletion aggravates lung injury and mortality through epithelial cells and fibroblasts in experimental murine sepsis
Source: Crit Care. 2023 Dec 13;27:490. doi: 10.1186/s13054-023-04777-z (PMC10717293; doi:10.1186/s13054-023-04777-z)
Supplement: Supplementary file 12 — Additional file 12. Table S3. Antibody list for flow cytometry. [file 13054_2023_4777_MOESM12_ESM.docx]

| Table S3. Antibody list for flow cytometry | | | |
| --- | --- | --- | --- |
| Antibody | Source | Conjugates | Catalog No. |
| CD11b | eBioscience | A780 | 47-0112-82 |
| CD11c | BD Biosciences | percp/cy5.5 | 560584 |
| Ly6G | eBioscience | FITC | 11-9668-82 |
| CD64 | eBioscience | PE | 12-0641-82 |
| MerTK | eBioscience | PE/Cy7 | 25-5751-82 |
| CD3 | eBioscience | e450 | 36-0031-85 |
| CD19 | eBioscience | Biotion | 13-0031-85 |
| NK1.1 | eBioscience | APC | 17-5941-82 |
| IA/IE | Biolegend | BV510 | 107636 |
| CD45 | eBioscience | A780 | 47-0451-82 |
| CD31 | eBioscience | PE/Cy7 | 25-0311-82 |
| CD326 | BD Pharmingen | PE | 563477 |
| PDGFRα | Biolegend | BV421 | 135923 |
| Ter119 | eBioscience | Biotion | 13-5921-85 |
| LYVE1 | eBioscience | Biotion | 13-0443-82 |
| IA/IE | eBioscience | Biotion | 13-5321-85 |
