# Supplementary material for: Interleukin-36 is overexpressed in human sepsis and IL-36 receptor deletion aggravates lung injury and mortality through epithelial cells and fibroblasts in experimental murine sepsis
Source: Crit Care. 2023 Dec 13;27:490. doi: 10.1186/s13054-023-04777-z (PMC10717293; doi:10.1186/s13054-023-04777-z)
Supplement: Supplementary file 13 — Additional file 13. Table S4. Expression of the top 5 differential genes on fibroblasts (GSE207651). [file 13054_2023_4777_MOESM13_ESM.docx]

| Table S4. Expression of the top 5 differential genes on fibroblasts (GSE207651). | | | | |
| --- | --- | --- | --- | --- |
| gene | p_val | avg_log2FC | pct.1 | pct.2 |
| Lcn2 | 1.05E-185 | 1.28311313 | 0.748 | 0.057 |
| S100a8 | 5.07E-178 | 1.042138701 | 0.926 | 0.359 |
| S100a9 | 9.11E-156 | 0.878587107 | 0.939 | 0.501 |
| Cfh | 1.27E-151 | 0.554307466 | 0.961 | 0.807 |
| Pcolce | 3.57E-150 | -0.906677547 | 0.17 | 0.651 |
